# Association of non-high-density lipoprotein cholesterol to high-density lipoprotein cholesterol ratio with all-cause mortality and cardio-cerebrovascular disease mortality in elderly patients with cardiovascular-kidney-metabolic syndrome stages 0–3: a cohort study

**DOI:** 10.3389/fnut.2026.1761136

**Published:** 2026-05-29

**Authors:** Suqi Xu, Weiquan Lin, Zhen Ling, Chuming Liao, Man Lv, Hui Liu, Zhoubin Zhang

**Affiliations:** 1 School of Public Health, Southern Medical University, Guangzhou, China; 2 Department of Basic Public Health Services, Guangzhou Center for Disease Control and Prevention (Guangzhou Health Supervision Institute), Guangzhou, China; 3 Institute of Public Health, Guangzhou Medical University and Guangzhou Center for Disease Control and Prevention, Guangzhou, China

**Keywords:** all-cause mortality, cardio-cerebrovascular disease mortality, ckm syndrome, ePWV, NHHR

## Abstract

**Background:**

Cardiovascular-kidney-metabolic (CKM) syndrome typically involves multiple metabolic dysregulations. The non-high-density lipoprotein cholesterol to high-density lipoprotein cholesterol ratio (NHHR), as a novel lipid metabolism biomarker, has been demonstrated to correlate with risks of mortality, metabolic syndrome, and chronic kidney disease. However, the relationship between NHHR and mortality risk remains unclear in elderly populations with CKM syndrome stages 0–3.

**Methods:**

This cohort study included 424,648 participants aged ≥ 65 years with CKM stages 0–3 from the Guangzhou Older Longitudinal Dynamic Health Cohort. Outcomes were all-cause and cardio-cerebrovascular disease mortality. We calculated the baseline NHHR, categorized participants by their quartiles, and employed Cox models and restricted cubic splines to examine the association between NHHR and mortality. Mediation analysis initially explored the potential role of estimated pulse wave velocity (ePWV) in these associations.

**Results:**

During a median follow-up of 4.93 years, 43,457 deaths occurred, including 19,590 from cardio-cerebrovascular diseases. After multivariable adjustment, both the lowest (Q1) and highest (Q4) NHHR quartiles showed higher risks compared with Q3 for all-cause mortality (Q1: HR: 1.13, 95% CI: 1.10–1.16; Q4: HR: 1.07, 95% CI: 1.04–1.10) and cardio-cerebrovascular disease mortality (Q1: HR: 1.09, 95% CI: 1.05–1.13; Q4: HR: 1.11, 95% CI: 1.07–1.16). Restricted cubic spline analyses suggested significant nonlinear (U-shaped) associations, with inflection points at 2.95 and 2.89 for the two outcomes. Mediation analysis suggested that ePWV accounted for approximately 40.16% of the association with cardio-cerebrovascular disease mortality.

**Conclusion:**

A U-shaped association was observed between NHHR and the risks of all-cause and cardio-cerebrovascular disease mortality in elderly individuals with CKM stages 0–3. These findings suggest NHHR may serve as an exploratory biomarker associated with mortality risk, but further validation is needed before clinical application.

**Clinical trial registration:**

Identifier (ChiCTR2400089945).

## 1 Introduction

Cardiovascular-kidney-metabolic (CKM) syndrome, as defined by the American Heart Association (AHA), is a complex systemic disorder characterized by the interplay among cardiovascular disease (CVD), chronic kidney disease (CKD), and metabolic disorders (1). It encompasses the interconnections between obesity, diabetes, CKD, and cardiovascular diseases. The pathophysiology of CKM syndrome is complex, involving metabolic disturbances such as insulin resistance, hyperglycemia, and dyslipidemia, as well as processes like chronic inflammation and oxidative stress (1, 2).

In recent years, the prevalence of CKM syndrome has been increasing. Evidence shows that the prevalence of CKM syndrome stage 3 has risen from 18.9 to 22.4% in men and from 13.9 to 15.2% in women (3), with a prevalence as high as 66.84% among individuals aged 60 and above (4). Additionally, research indicated that 32.7% of U.S. adults have at least one CKM-related condition, while 14.4% have two or more such conditions. Compared to those without CKM syndrome, affected patients have a significantly higher risk of all-cause and cardiovascular mortality, a risk that escalates with the number of CKM-related conditions (5). In China, CKM syndrome is also highly prevalent. Approximately 80% of adults are in stage 1 or higher, and 23.6% are in an advanced stage (stage 4). Moreover, advanced CKM syndrome is closely associated with an increased risk of all-cause, cardiovascular, and non-cardiovascular mortality (6). Multiple studies have further indicated that a higher CKM syndrome stage correlates with a greater mortality risk (6, 7). The high prevalence and mortality of CKM syndrome not only threaten individual health but also impose a substantial burden on global public health and society (1, 8). Notably, the AHA emphasizes the importance of early screening for asymptomatic individuals in the early stages of CKM syndrome (1). Consequently, growing evidence underscores the necessity for a unified strategy to prevent disease progression in individuals with CKM syndrome stages 0–3.

Dyslipidemia is common in patients with CKM syndrome and contributes to an increased risk of cardiovascular disease and related mortality (1, 9). However, conventional lipid parameters, such as low-density lipoprotein cholesterol (LDL-C) and high-density lipoprotein cholesterol (HDL-C), have limitations in risk assessment for multi-system disorders, like CKM syndrome (9–11). The non-high-density lipoprotein cholesterol to high-density lipoprotein cholesterol ratio (NHHR) has recently emerged as a promising novel lipid marker. NHHR reflects overall lipid metabolic health and has demonstrated predictive value for mortality, metabolic syndrome, and chronic kidney disease (12, 13). Despite this, existing studies on NHHR have predominantly been conducted in general populations or in individuals with specific cardiometabolic conditions. For instance, analyses based on the NHANES database have demonstrated significant associations between NHHR and both all-cause and cardiovascular mortality in the general US adult population (13). In addition, similar associations have been reported in patients with hypertension (14) and in those with diabetes or prediabetes (15). Furthermore, emerging evidence from cohort studies involving middle-aged and elderly populations and individuals with metabolic disorders has also suggested that elevated NHHR is associated with an increased risk of cardiovascular mortality (16, 17). However, most prior studies have focused on general populations or single disease conditions, with limited evidence in populations characterized by overlapping metabolic, renal, and cardiovascular abnormalities. CKM syndrome provides an integrated framework to describe this clustering of cardiometabolic and renal dysfunction, and may offer a more relevant context for examining lipid-related risk markers.

Therefore, this study aimed to investigate the association between NHHR and the risks of all-cause and cardio-cerebrovascular disease mortality in elderly individuals with CKM stages 0–3. Specifically, we sought to evaluate whether NHHR is associated with mortality outcomes within the CKM framework and to provide additional evidence in an elderly population.

## 2 Methods

### 2.1 Study design and population

This cohort study utilized data from the Guangzhou Older Longitudinal Dynamic Health (GOLD-Health) Cohort (18). The GOLD-Health cohort was a large-scale prospective study investigating health dynamics among residents of Guangzhou, China. The study protocol was reviewed and approved by the Ethics Committee of the Medical Ethics Research Center of the Guangzhou Center for Disease Control and Prevention (Approval Number: GZCDC-ECHR-2023P0081). All participants provided written informed consent before enrollment.

**Figure 1:**
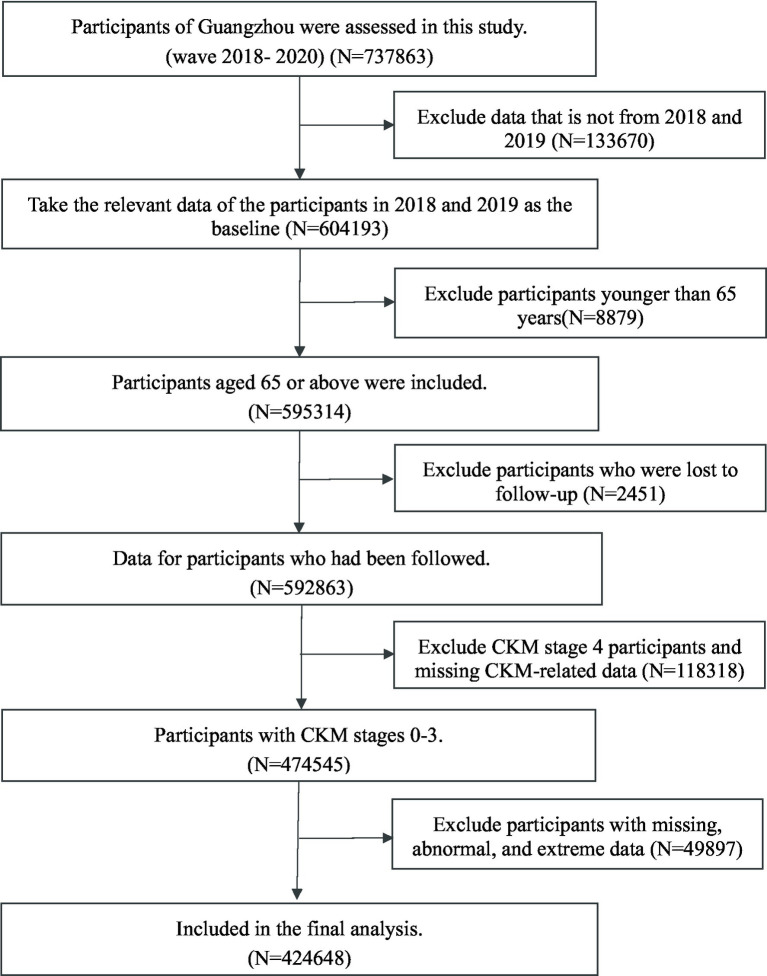
Flowchart of study population.

The baseline population consisted of participants enrolled in 2018 and 2019, who were then prospectively followed until December 31, 2023. From an initial pool of 737,863 individuals recruited between 2018 and 2020, we established a baseline sample of 604,193 participants. During the screening process, we excluded participants who were aged < 65 years at baseline (*N* = 8,879), those in CKM syndrome stage 4 or with missing data relating to CKM syndrome (*N* = 118,318), those lost to follow-up (*N* = 2,451), and those with missing, abnormal, or extreme data (*N* = 49,897). After these exclusions, the final analytical sample comprised 424,648 participants (Figure 1). All participants underwent comprehensive baseline surveys and health assessments, including face-to-face questionnaire interviews, physical examinations, and laboratory tests. Data collection was performed by trained clinicians following standardized protocols. Venous blood samples were collected from all participants under fasting conditions. Participants were instructed to fast for 12 h before blood collection (18). All biochemical measurements were conducted following standardized laboratory procedures to ensure accuracy and consistency.

### 2.2 Calculation of NHHR

NHHR was calculated by the following formula (19): NHHR = non-HDL-C/HDL-C, non-HDL-C = TC − HDL-C, where TC is total cholesterol.

### 2.3 Definition of CKM syndrome stages 0 to 3

CKM syndrome stages were defined according to the AHA criteria (1). Participants were categorized into CKM syndrome stages 0–3 as follows: Stage 0: No CKM syndrome risk factors. Stage 1: Excess or dysfunctional adiposity. Stage 2: Individuals with metabolic risk factors (hypertriglyceridemia, hypertension, MetS, diabetes), or CKD. Stage 3: Subclinical CVD in CKM syndrome. The estimated Glomerular Filtration Rate (eGFR) was calculated using serum creatinine (Scr) (20). Classification of CKD stages according to the Kidney Disease Improving Global Outcomes (KDIGO) guidelines (1). Due to the lack of imaging-based measures of subclinical CVD in this study, subclinical CVD was operationally defined as either high-risk CKD (KDIGO stage G4 or G5) or a predicted 10-year CVD risk of ≥ 20%, as assessed using the Framingham risk score (21) (Supplementary Tables S1–S4).

Participants with CKM syndrome stage 4 were excluded at baseline. A detailed summary of the staging criteria for CKM syndrome stages 0–4 is provided in Supplementary Table S5, and the diagnostic definitions for all component conditions (e.g., overweight, diabetes, hypertension, CKD) are outlined in Supplementary Table S6. In cases where participants met criteria for multiple CKM syndrome stages, a hierarchical classification approach was applied, whereby individuals were assigned to the highest applicable stage.

### 2.4 Definition of outcomes

Participants were followed from their date of enrollment until December 31, 2023. The primary endpoints were all-cause mortality and cardio-cerebrovascular disease mortality events. Mortality data were obtained from the Guangzhou City Cause-of-Death Registration and Reporting Information System. The specific causes of death were classified according to the International Classification of Diseases, Tenth Revision (ICD-10). Deaths from cardio-cerebrovascular diseases were defined by ICD-10 codes I00-I99 (22). All-cause mortality was defined as death from any cause.

### 2.5 Covariates

To assess multicollinearity and select covariates, we used the Variance Inflation Factor (VIF). A VIF value greater than 5 is generally considered to indicate significant multicollinearity. We considered a set of potential covariates including sex, age groups(grouped as 65–69, 70–79, ≥80 years), education level(illiterate, primary school, junior high school, senior high school, or university and above), marital status (married or other), physical activity (PA, grouped as no exercise, occasional, weekly or more, daily), body mass index (BMI, categorized as <18.5, 18.5–24.0, or ≥ 24.0 kg/m^2^), smoking status (yes/no), and drinking status (yes/no). The history of medication for hypertension and diabetes (yes/no) was also included. The VIF for all these covariates was below 5, and therefore, all were retained in the final multivariate model (Supplementary Table S7).

### 2.6 Statistical analyses

To ensure data quality and robustness, outliers were identified and excluded prior to analysis. Observations beyond ± 3 standard deviations (SD) from the mean were defined as outliers. This approach is commonly used in epidemiological studies to reduce the influence of extreme values and potential measurement errors in large cohort datasets (23, 24). To assess potential selection bias due to exclusions, baseline characteristics were compared between included and excluded participants using the same statistical methods. Standardized mean differences (SMD) were calculated to quantify group differences, with an absolute SMD > 0.10 indicating a meaningful imbalance (Supplementary Table S8). Descriptive statistics were presented according to variable type. Normally distributed continuous variables were expressed as mean ± SD and compared using analysis of variance (ANOVA), while non-normally distributed variables were described as median (interquartile range, IQR) and compared using the Kruskal–Wallis test. Categorical variables were summarized as counts and percentages and compared using the chi-square test. NHHR was analyzed as both a continuous and categorical variable. In the restricted cubic spline (RCS) analysis, NHHR was included as a continuous variable in the model to explore the potential non-linear association between it and the risk of death. NHHR was additionally categorized into quartiles for descriptive analyses. In the categorical analyses, the third quartile (Q3) was used as a reference category to facilitate comparisons across exposure levels around the range of the lowest estimated risk suggested by the spline curves. Using Q3 as the reference allowed comparisons of both lower and higher NHHR levels against a mid-range category, which may better reflect the central tendency of the exposure distribution. The proportional hazards assumption was visually assessed using scaled Schoenfeld residual plots, with primary reliance on the horizontality of the smoothed curves rather than formal *p* values, given the high sensitivity of conventional tests to minor fluctuations in this large cohort (Supplementary Figure S1). Cox proportional hazards regression models were used to estimate the hazard ratio (HR) and 95% confidence interval (CI). Three models were constructed: Model 1: unadjusted; Model 2: adjusted for sex, age groups; and Model 3: further adjusted for marital status, BMI, education level, smoking status, drinking status, PA, antihypertensive medication, and antidiabetic medication. To further explore whether the nonlinear associations between NHHR and mortality differed across disease severity, restricted cubic spline analyses stratified by CKM stages 0–3 were performed. Subgroup and interaction analyses were conducted according to sex, age, smoking status, alcohol consumption, and CKM stage.

To verify the robustness of our findings, we conducted six sensitivity analyses. In sensitivity analysis 1, we applied inverse probability of censoring weighting (IPCW) to further address potential selection bias due to missing data and censoring. Robustness was assessed by evaluating the consistency between the IPCW-weighted estimates and those from the complete-case analysis. In sensitivity analysis 2 to assess the robustness of using the Q3 of NHHR as the reference group, the first quartile (Q1) was used as the reference group in the Cox regression models. In sensitivity analysis 3, participants with cancer at baseline were excluded to reduce potential confounding from severe underlying diseases. In sensitivity analysis 4, participants with a follow-up duration of less than 2 years were excluded to minimize the impact of reverse causation and early death events. In sensitivity analysis 5, lipid-lowering medication use was additionally included as a covariate in the fully adjusted model to reduce potential confounding from pharmacological interventions affecting lipid levels and their associations with mortality. In sensitivity analysis 6, alternative BMI classifications based on the World Health Organization (WHO) criteria (BMI ≥ 25 kg/m^2^ and ≥ 30 kg/m^2^) were additionally included as covariates in the fully adjusted model to reduce potential confounding arising from BMI categorization differences.

To investigate the underlying mechanisms, we performed mediation analysis following VanderWeele’s framework for survival data (25). This approach allowed us to calculate the total, direct, and indirect effects. We also determined the proportion of mediation. To ensure robustness, we generated 95% CI based on 1,000 bootstrap resamples. Given that arterial stiffness is a recognized predictor of adverse cardiovascular events and mortality (26, 27), we used the estimated pulse wave velocity (ePWV) as a potential mediator in the relationship between NHHR and mortality risks. The ePWV was calculated using the following formula (28): ePWV = 9.587–0.402 × age + 4.560 × 10^−3^ × age^2^–2.621 × 10^−5^ × age^2^ × MBP + 3.176 × 10^−3^ × age × MBP—1.832 × 10^−2^ × MBP, where MBP (mean blood pressure) = DBP + 0.4 × [SBP (Systolic blood pressure)—DBP (Diastolic blood pressure)].

All statistical analyses were performed using *R* software (version 4.5.0), and a two-sided *p* value < 0.05 was considered statistically significant.

## 3 Results

### 3.1 Characteristics of the participants

A total of 424,648 participants were included in this study, with a mean age of 72.27 ± 6.48 years. At baseline, 39.9% were male and 60.1% were female. Table 1 presents baseline characteristics across NHHR quartiles (Q1: ≤2.11; Q2: 2.11–2.76; Q3: 2.76–3.49; Q4: >3.49). Participants in the highest quartiles (Q4) had higher proportions of overweight/obesity and higher levels of Scr, fasting blood glucose (FBG), triglyceride (TG), TC, and LDL-C. They also had a higher prevalence of hypertension and diabetes, and a greater proportion of antihypertensive and hypoglycemic medication use, as well as a higher proportion of CKM stage 3.

**Table 1:** Baseline characteristics according to NHHR quartiles.

Characteristics	Quartiles of NHHR					*p-*value
	Overall	Q1 (≤ 2.11)	Q2 (2.11–2.76)	Q3 (2.76–3.49)	Q4 (> 3.49)	
*N* (%)	424,648	106,266	106,173	106,158	106,051	
Sex (%)						<0.001
Male	169,486 (39.9)	41,610 (39.2)	40,898 (38.5)	41,733 (39.3)	45,245 (42.7)	
Female	255,162 (60.1)	64,656 (60.8)	65,275 (61.5)	64,425 (60.7)	60,806 (57.3)	
Age, (mean ± SD), years	72.27 ± 6.48	72.97 ± 6.88	72.29 ± 6.47	71.96 ± 6.28	71.87 ± 6.20	<0.001
Age groups, (%), years						<0.001
65–69	185,524 (43.7)	42,875 (40.3)	46,264 (43.6)	48,092 (45.3)	48,293 (45.5)	
70–79	172,520 (40.6)	43,093 (40.6)	43,136 (40.6)	43,126 (40.6)	43,165 (40.7)	
≥ 80	66,604 (15.7)	20,298 (19.1)	16,773 (15.8)	14,940 (14.1)	14,593 (13.8)	
Marital status (%)						<0.001
Married	370,754 (87.3)	92,045 (86.6)	92,409 (87.0)	92,963 (87.6)	93,337 (88.0)	
Other^*^	53,894 (12.7)	14,221 (13.4)	13,764 (13.0)	13,195 (12.4)	12,714 (12.0)	
BMI (%), kg/m^2^						<0.001
Underweight	20,308 (4.8)	10,342 (9.7)	5,201 (4.9)	3,044 (2.9)	1721 (1.6)	
Normal	217,866 (51.3)	62,898 (59.2)	57,515 (54.2)	51,338 (48.4)	46,115 (43.5)	
Overweight or obese	186,474 (43.9)	33,026 (31.1)	43,457 (40.9)	51,776 (48.8)	58,215 (54.9)	
Education level (%)						<0.001
Illiterate	45,296 (10.7)	11,954 (11.2)	11,548 (10.9)	11,018 (10.4)	10,776 (10.2)	
Primary school	135,660 (31.9)	33,367 (31.4)	33,610 (31.7)	33,978 (32.0)	34,705 (32.7)	
Middle school	112,906 (26.6)	27,724 (26.1)	27,550 (25.9)	28,216 (26.6)	29,416 (27.7)	
High school	38,324 (9.0)	9,530 (9.0)	9,476 (8.9)	9,241 (8.7)	10,077 (9.5)	
University or higher	92,462 (21.8)	23,691 (22.3)	23,989 (22.6)	23,705 (22.3)	21,077 (19.9)	

Characteristics	Quartiles of NHHR					*p-*value
PA (%)						<0.001
No exercise	118,870 (28.0)	32,115 (30.2)	29,648 (27.9)	28,890 (27.2)	28,217 (26.6)	
Occasional	27,182 (6.4)	6,232 (5.9)	6,569 (6.2)	6,820 (6.4)	7,561 (7.1)	
Weekly or more	104,704 (24.7)	26,670 (25.1)	26,581 (25.0)	25,803 (24.3)	25,650 (24.2)	
Daily	173,892 (40.9)	41,249 (38.8)	43,375 (40.9)	44,645 (42.1)	44,623 (42.1)	
Smoking status (%)						<0.001
No	361,339 (85.1)	91,848 (86.4)	91,191 (85.9)	90,406 (85.2)	87,894 (82.9)	
Yes	63,309 (14.9)	14,418 (13.6)	14,982 (14.1)	15,752 (14.8)	18,157 (17.1)	
Drinking status (%)						<0.001
No	400,132 (94.2)	99,671 (93.8)	100,039 (94.2)	100,244 (94.4)	100,178 (94.5)	
Yes	24,516 (5.8)	6,595 (6.2)	6,134 (5.8)	5,914 (5.6)	5,873 (5.5)	
Hypertension (%)						<0.001
No	147,034 (34.6)	40,941 (38.5)	37,965 (35.8)	35,358 (33.3)	32,770 (30.9)	
Yes	277,614 (65.4)	65,325 (61.5)	68,208 (64.2)	70,800 (66.7)	73,281 (69.1)	
Diabetes (%)						<0.001
No	335,982 (79.1)	86,747 (81.6)	85,472 (80.5)	83,594 (78.7)	80,169 (75.6)	
Yes	88,666 (20.9)	19,519 (18.4)	20,701 (19.5)	22,564 (21.3)	25,882 (24.4)	
Cancer (%)						0.109
No	418,697 (98.6)	104,746 (98.6)	104,715 (98.6)	104,730 (98.7)	104,506 (98.5)	
Yes	5,951 (1.4)	1,520 (1.4)	1,458 (1.4)	1,428 (1.3)	1,545 (1.5)	
Antihypertensive medication (%)						<0.001
No	245,426 (57.8)	64,686 (60.9)	62,637 (59.0)	60,266 (56.8)	57,837 (54.5)	
Yes	179,222 (42.2)	41,580 (39.1)	43,536 (41.0)	45,892 (43.2)	48,214 (45.5)	

Characteristics	Quartiles of NHHR					*p-*value
Antidiabetic medication (%)						<0.001
No	370,923 (87.3)	94,092 (88.5)	93,291 (87.9)	92,495 (87.1)	91,045 (85.9)	
Yes	53,725 (12.7)	12,174 (11.5)	12,882 (12.1)	13,663 (12.9)	15,006 (14.1)	
CKM stages (%)						<0.001
0	22,271 (5.2)	11,203 (10.5)	6,432 (6.1)	3,311 (3.1)	1,325 (1.2)	
1	29,037 (6.8)	11,270 (10.6)	9,429 (8.9)	6,025 (5.7)	2,313 (2.2)	
2	150,429 (35.4)	44,546 (41.9)	41,457 (39.0)	37,269 (35.1)	27,157 (25.6)	
3	222,911 (52.5)	39,247 (36.9)	48,855 (46.0)	59,553 (56.1)	75,256 (71.0)	
Height, (median, IQR), m	1.56 (1.50,1.63)	1.56 (1.50, 1.62)	1.56 (1.50, 1.62)	1.56 (1.50, 1.62)	1.56 (1.51, 1.63)	<0.001
Weight, (median, IQR), kg	57.70 (51.00, 64.50)	55.00 (48.40, 61.50)	57.00 (50.60, 63.75)	58.50 (52.20, 65.15)	60.00 (53.90, 67.00)	<0.001
WC, (median, IQR), cm	83.50 (78.00, 89.00)	80.50 (75.00, 86.00)	83.00 (77.50, 88.50)	84.00 (79.00, 90.00)	85.50 (80.00, 91.00)	<0.001
DBP, (median, IQR), mmHg	78.00 (72.00, 84.00)	77.00 (71.00, 82.00)	78.00 (72.00, 84.00)	78.00 (73.00, 84.00)	79.00 (73.00, 84.00)	<0.001
SBP, (median, IQR), mmHg	135.00 (126.00, 147.00)	134.00 (124.00, 145.00)	135.00 (126.00, 146.00)	136.00 (127.00, 148.00)	136.00 (128.00, 148.00)	<0.001
Scr, (median, IQR), mg/dl	0.88 (0.74, 1.04)	0.86 (0.73, 1.01)	0.87 (0.74, 1.03)	0.88 (0.74, 1.04)	0.91 (0.77, 1.08)	<0.001
FBG, (median, IQR), mg/dl	97.00 (89.00, 109.00)	95.00 (87.00, 106.00)	96.00 (88.00, 108.00)	98.00 (89.00, 110.00)	100.00 (90.00, 113.00)	<0.001
TG, (median, IQR), mg/dl	123.00 (90.00, 170.00)	90.00 (70.00, 120.00)	112.00 (87.00, 147.00)	135.00 (104.00, 177.00)	168.00 (128.00, 228.00)	<0.001

Characteristics	Quartiles of NHHR					*p-*value
TC, (median, IQR), mg/dl	208.00 (181.00, 236.00)	184.00 (159.00, 209.00)	203.00 (179.00, 227.00)	214.00 (190.00, 239.00)	231.00 (205.00, 258.00)	<0.001
LDL-C, (median, IQR), mg/dl	124.00 (102.00, 148.00)	101.00 (82.00, 119.00)	120.00 (102.00, 139.00)	132.00 (113.00, 153.00)	148.00 (125.00, 171.00)	<0.001
HDL-C, (median, IQR), mg/dl	55.00 (46.00, 65.00)	70.00 (60.00, 80.00)	59.00 (52.00, 66.00)	52.00 (46.00, 58.00)	44.00 (39.00, 50.00)	<0.001

Data are summarized as *n* (%), mean ± SD, (median, IQR). SD, standard deviation; IQR, Interquartile range; BMI, Body mass index; PA, Physical activity; CKM stages, Cardiovascular-kidney-metabolic syndrome stages; WC, Waist circumference; DBP, Diastolic blood pressure; SBP, Systolic blood pressure; Scr, Serum creatinine; FBG, Fasting blood glucose; TG, Triglyceride; TC, Total cholesterol; LDL-C, Low-density lipoprotein cholesterol; HDL-C, High-density lipoprotein cholesterol. Other*: unmarried, widowed, divorced.

### 3.2 Cox regression analysis of the relationship between NHHR and mortality in elderly participants with CKM stages 0–3

**Figure 2:**
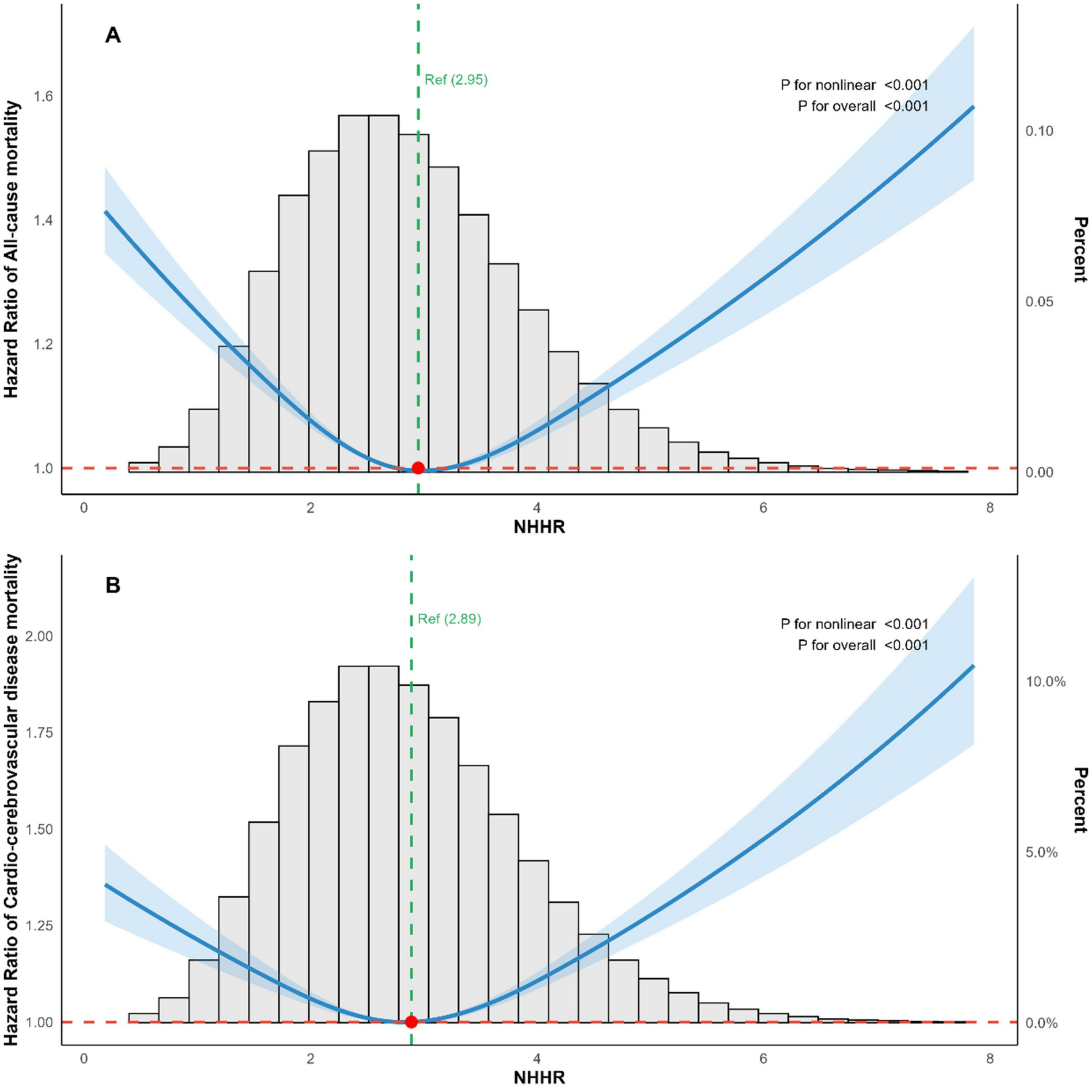
Restrictive cubic splines plot between NHHR and mortality in elderly participants with CKM stages 0–3. Restricted cubic splines were used to evaluate the hypothesis of potential nonlinear relationships between NHHR and all-cause mortality **(A)** and cardio-cerebrovascular disease mortality **(B)** in elderly participants with CKM stages 0–3. The NHHRs of 2.95 for A and 2.89 for B were chosen as reference estimates for each hazard ratio (HR). The analysis was adjusted for sex, age groups, marital status, BMI, education level, smoking status, drinking status, PA, antihypertensive medication, and antidiabetic medication. BMI: Body mass index, PA: Physical activity, CKM stages: Cardiovascular-kidney-metabolic syndrome stages. The restricted cubic spline analysis suggested a U-shaped association between NHHR and mortality risks, with relatively lower risk estimates observed around the third quartile (Q3). Accordingly, Q3 was used as the reference category for illustrative comparisons in the categorical analyses.

During a median follow-up of 4.93 years, 43,457 deaths occurred, among which 19,590 deaths were due to cardio-cerebrovascular disease. The associations between NHHR and mortality are shown in Table 2. Based on the observed U-shaped association in the RCS analysis (Figure 2), with a relatively lower risk estimated around the third quartile (Q3), Q3 was selected as the reference category in categorical analyses to facilitate comparisons across exposure levels. In the fully adjusted model (Model 3), compared with Q3, both Q1 and Q4 were associated with higher risks of all-cause mortality. The all-cause mortality risk increased by 13% in Q1 (HR: 1.13, 95% CI: 1.10–1.16, *p* < 0.001) and by 7% in Q4 (HR: 1.07, 95% CI: 1.04–1.10, *p* < 0.001). For cardio-cerebrovascular disease mortality, similar associations were observed (Q1: HR 1.09, 95% CI 1.05–1.13, *p* < 0.001; Q4: HR 1.11, 95% CI 1.07–1.16, *p* < 0.001).

**Table 2:** The association between NHHR and mortality in elderly participants with CKM stages 0–3.

Variables	Number of deaths	Model 1, HR (95% CI) *p*-value	Model 2, HR (95% CI) *p*-value	Model 3, HR (95% CI) *p-*value
All-cause mortality				
Q3	9,600	Ref	Ref	Ref
Q1	13,129	1.40 (1.36–1.44) < 0.001	1.22 (1.19–1.26) < 0.001	1.13 (1.10–1.16) < 0.001
Q2	10,543	1.10 (1.07–1.14) < 0.001	1.05 (1.02–1.08) < 0.001	1.02 (0.99–1.05) 0.246
Q4	10,185	1.06 (1.03–1.09) < 0.001	1.06 (1.03–1.09) < 0.001	1.07 (1.04–1.10) < 0.001
Cardio-cerebrovascular disease mortality				
Q3	4,363	Ref	Ref	Ref
Q1	5,762	1.35 (1.30–1.41) < 0.001	1.16 (1.11–1.20) < 0.001	1.09 (1.05–1.13) < 0.001
Q2	4,669	1.08 (1.03–1.12) < 0.001	1.02 (0.98–1.06) 0.446	0.99 (0.95–1.03) 0.577
Q4	4,796	1.10 (1.06–1.15) < 0.001	1.10 (1.06–1.15) < 0.001	1.11 (1.07–1.16) < 0.001

HR, hazard ratio, 95%; CI, 95% confidence interval.

Model 1: crude model. Model 2: adjusted for gender and age groups. Model 3: adjusted for sex, age groups, marital status, BMI, education level, smoking status, drinking status, PA, antihypertensive medication, and antidiabetic medication.

BMI, Body mass index; PA, Physical activity; CKM stages, Cardiovascular-kidney-metabolic syndrome stages.

When analyzed as a continuous variable, each one-unit increase in NHHR was associated with a 3% higher risk of cardio-cerebrovascular disease mortality (HR: 1.03, 95% CI: 1.01–1.04, *p* < 0.001) in the fully adjusted model, but it is not related to the risk of all-cause mortality (*p* = 0.146) (Table 3).

**Table 3:** Threshold effect analysis of NHHR on mortality in elderly participants with CKM stages 0–3.

^a^Adjusted models	Adjusted HR (95% CI), *p*-value
All-cause mortality	
Model 1: standard cox regression model	0.99 (0.98–1.00) 0.146
Model 2: segmented cox proportional hazards model	
Inflection point	2.95
NHHR<2.95	0.88 (0.86–0.90) < 0.001
NHHR≥2.95	1.10 (1.08–1.12) < 0.001
*p* for log-likelihood ratio test^b^	*p* < 0.001
Cardio-cerebrovascular disease mortality	
Model 1: standard cox regression model	1.03 (1.01–1.04) < 0.001
Model 2: segmented cox proportional hazards model	
Inflection point	2.89
NHHR<2.89	0.90 (0.86–0.93) < 0.001
NHHR≥2.89	1.14 (1.11–1.17) < 0.001
*p* for log-likelihood ratio test^b^	*p* < 0.001

HR, hazard ratio 95% CI, 95% confidence interval.

^a^Adjusted models: sex, age groups, marital status, BMI, education level, smoking status, drinking status, PA, antihypertensive medication, and antidiabetic medication.

^b^Model 2 compared to Model 1.

BMI, Body mass index; PA, Physical activity; CKM stages, Cardiovascular-kidney-metabolic syndrome stages.

### 3.3 RCS and threshold effect analysis

The dose–response relationship between NHHR and mortality was modeled using RCS (Figure 2). The RCS analysis revealed a significant non-linear, U-shaped association between NHHR and both all-cause and cardio-cerebrovascular disease mortality (*p* for overall < 0.001; *p* for non-linearity < 0.001). Specifically, as NHHR increased, the risks of all-cause and cardio-cerebrovascular disease mortality initially decreased, reached a nadir, and then subsequently increased.

Using the “segmented” package, we identified the inflection points for the relationships of NHHR with all-cause and cardio-cerebrovascular disease mortality, which were 2.95 and 2.89, respectively. A piecewise linear regression model was further applied to quantify the associations on both sides of these inflection points (Table 3). Below the inflection point, NHHR was negatively associated with mortality. For each one-unit increase in NHHR, the risk of all-cause mortality decreased by 12% (HR: 0.88, 95% CI: 0.86–0.90, *p* < 0.001), and the risk of cardio-cerebrovascular disease mortality decreased by 10% (HR: 0.90, 95% CI: 0.86–0.93, *p* < 0.001). Conversely, above the inflection point, NHHR was positively associated with mortality. For each one-unit increase in NHHR, the risk of all-cause mortality increased by 10% (HR: 1.10, 95% CI: 1.08–1.12, *p* < 0.001), and the risk of cardio-cerebrovascular disease mortality increased by 14% (HR: 1.14, 95% CI: 1.11–1.17, *p* < 0.001).

Stratified RCS analyses across CKM stages showed consistent nonlinear patterns (Supplementary Figure S1). The location of the nadir varied across CKM stages, suggesting potential heterogeneity in the shape of the association. However, a consistent U-shaped association between NHHR and both all-cause and cardio-cerebrovascular disease mortality was observed across all CKM stages after adjustment for potential confounders (*p* for nonlinear < 0.05).

### 3.4 Subgroup and interaction analyses

**Figure 3:**
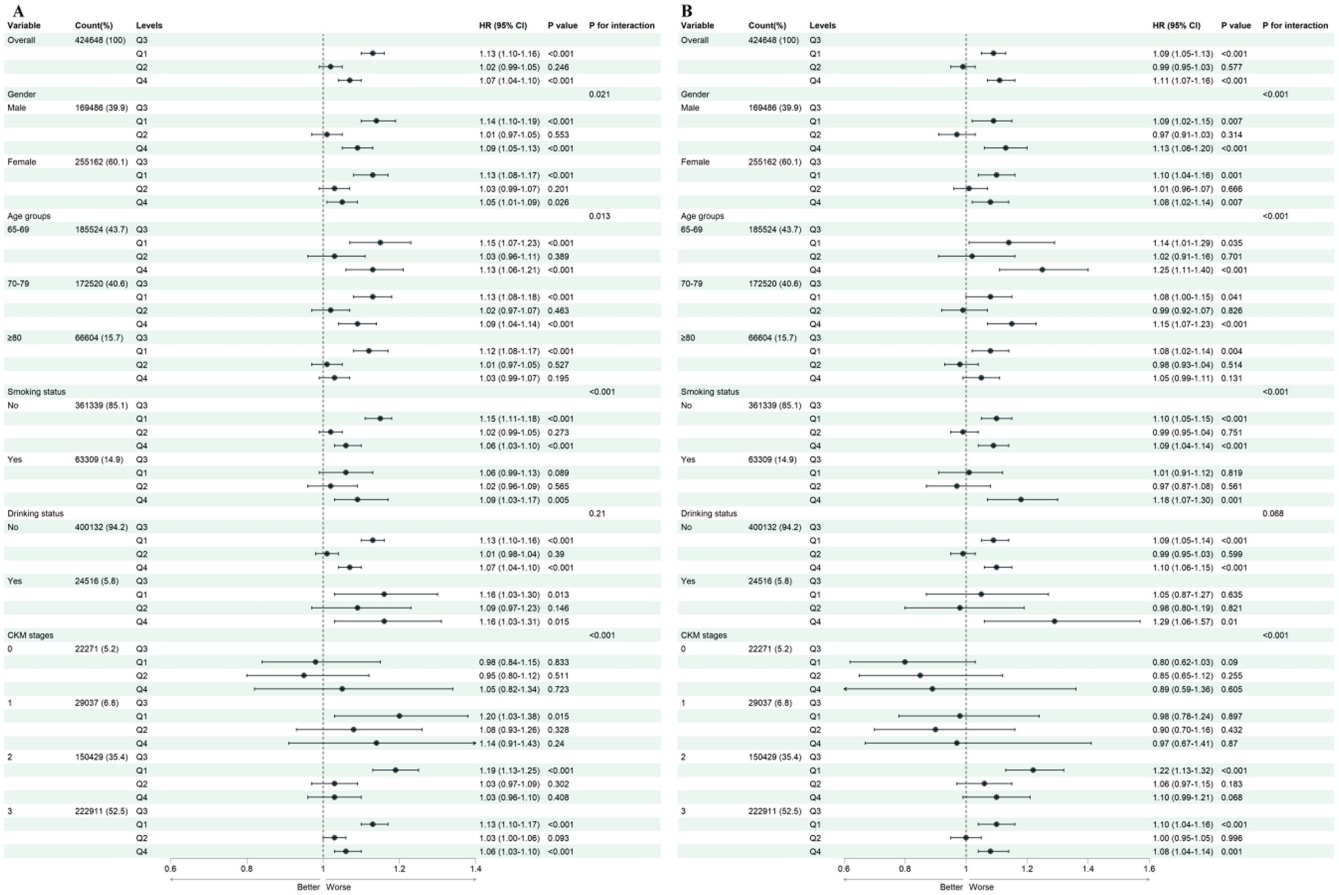
Subgroup analysis of the association between NHHR and mortality in the elderly population with CKM stages 0–3. **(A)** Subgroup analysis of the association between NHHR and all-cause mortality risk in the elderly population with CKM stages 0–3. **(B)** Subgroup analysis of the association between NHHR and cardio-cerebrovascular disease mortality risk in the elderly population with CKM stages 0–3. The HR was calculated using the Cox proportional hazards model with the adjustments including sex, age groups, marital status, BMI, education level, smoking status, drinking status, PA, antihypertensive medication, and antidiabetic medication. HR: hazard ratio, 95% CI: 95% confidence interval. BMI: body mass index, PA: physical activity, CKM stages: cardiovascular-kidney-metabolic syndrome stages.

To investigate the relationship between NHHR and the risk of all-cause mortality and cardio-cerebrovascular disease mortality, subgroup analyses were conducted. The results are detailed in Figure 3, Supplementary Table S9, and Supplementary Table S10.

Subgroup analyses revealed that both the lowest (Q1) and highest (Q4) NHHR were significant risk factors for all-cause and cardio-cerebrovascular disease mortality. These associations remained largely consistent across most subgroups. Significant interaction effects were observed for sex, age groups, smoking status, and CKM stages (*P* for interaction <0.05), indicating that the magnitude of the risk associated with NHHR levels varied across these strata. Notably, within the CKM stage 3 subgroup, both Q1 and Q4 were independently associated with elevated mortality.

**Figure 4:**
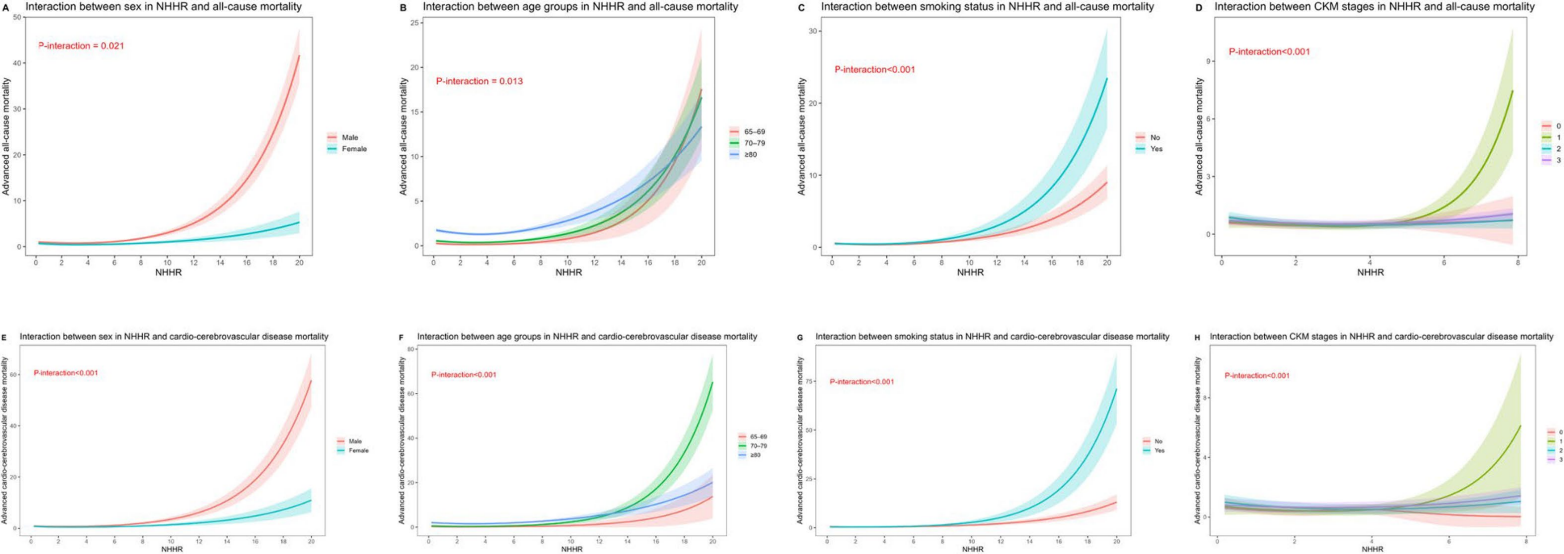
Subgroup interaction effect plots. **(A)** Interaction between sex in NHHR and all-cause mortality; **(B)** Interaction between age groups in NHHR and all-cause mortality; **(C)** Interaction between smoking status in NHHR and all-cause mortality; **(D)** Interaction between CKM stages in NHHR and all-cause mortality; **(E)** Interaction between sex in NHHR and cardio-cerebrovascular disease mortality; **(F)** Interaction between age groups in NHHR and cardio-cerebrovascular disease mortality; **(G)** Interaction between smoking status in NHHR and cardio-cerebrovascular disease mortality; **(H)** Interaction between CKM stages in NHHR and cardio-cerebrovascular disease mortality. CKM stages: Cardiovascular-kidney-metabolic syndrome stages.

Figure 4 further illustrates these interactions. For all-cause mortality, there is a non-linear association between NHHR and the risk of death, and this association is significantly modified by multiple factors: for males, those aged 80 or above, smokers, and those in CKM stage 3, the risk increases more steeply at higher levels of NHHR. The mortality rate due to cardiovascular and cerebrovascular diseases shows a similar pattern. In summary, both too low and too high levels of NHHR are associated with an increased risk of death, and this association is significantly influenced by demographic and clinical factors.

### 3.5 Sensitivity analyses

Across six sensitivity analyses (Supplementary Tables S11–S16), the associations between NHHR and mortality remained generally consistent.

Baseline differences between included and excluded participants were observed, with several variables exceeding the SMD threshold of 0.10, suggesting potential non-random missingness (Supplementary Table S8). Nevertheless, the consistency between IPCW and complete-case analyses supports the robustness of the findings (Supplementary Table S11).

When using Q1 as the reference group, the inverse associations remained stable, with Q3 exhibiting the lowest mortality risk, further supporting the robustness of the chosen reference group (Supplementary Table S12). Results were robust after excluding participants with cancer at baseline, excluding early follow-up deaths, adjusting for lipid-lowering medication, and using alternative BMI classifications (Supplementary Tables S13–S16). Overall, these analyses suggest that the observed associations are relatively robust to different model specifications and sensitivity assumptions.

### 3.6 Mediation analysis

**Figure 5:**
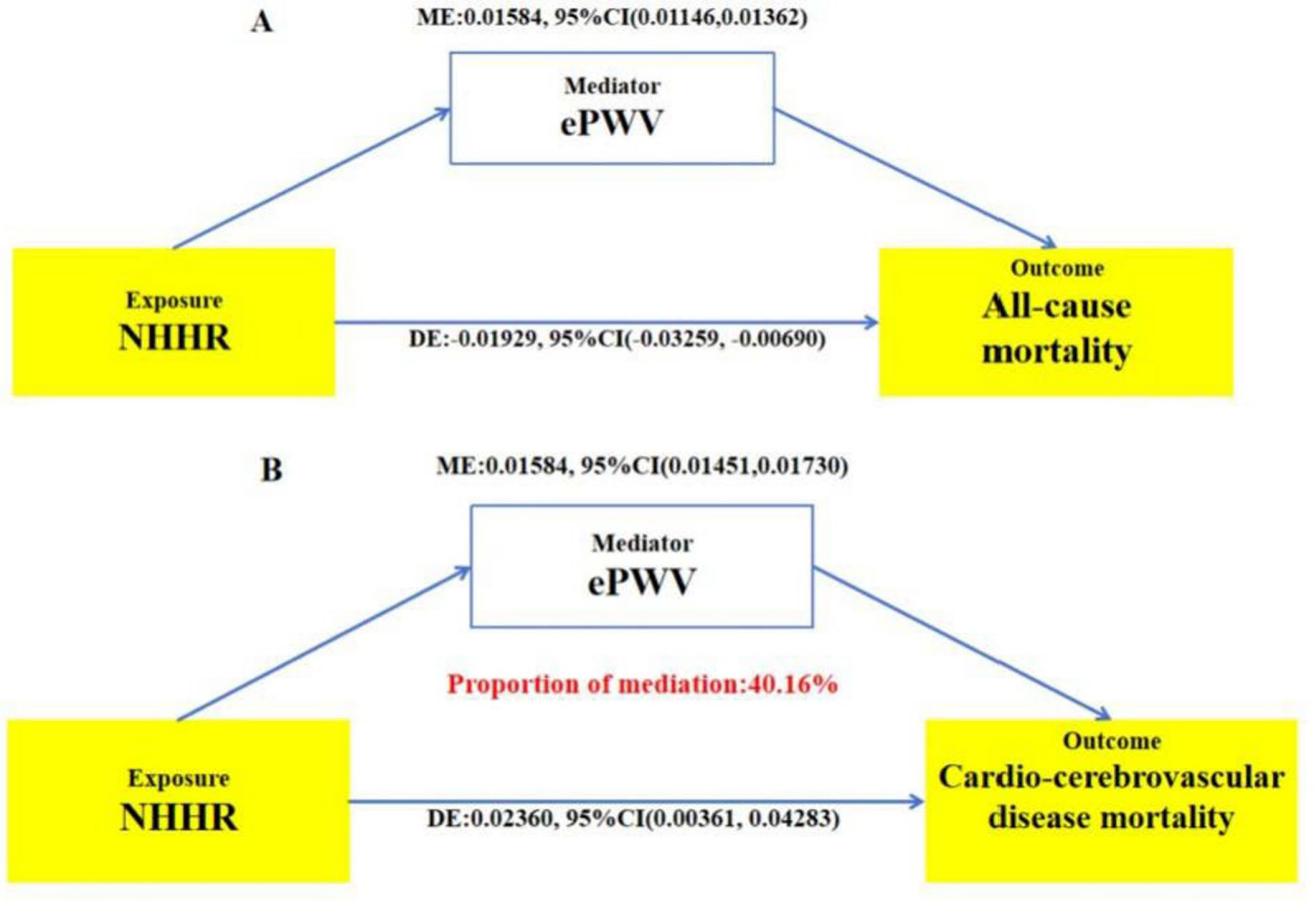
Mediation of the association between NHHR and mortality by ePWV. **(A)** Mediation of the association between NHHR and all-cause mortality risk by ePWV. **(B)** Mediation of the association between NHHR and cardio-cerebrovascular disease mortality risk by ePWV. Adjusted for sex, age groups, marital status, BMI, education level, smoking status, drinking status, PA, antihypertensive medication, and antidiabetic medication. BMI, Body mass index; PA, Physical activity.

Mediation analysis based on Vander Weele’s framework suggested that ePWV may partially explain the association between NHHR and cardio-cerebrovascular disease mortality (Figure 5). After adjusting for confounding factors, the proportion mediated was estimated at 40.16%. In contrast, no significant mediating effect of ePWV was observed for all-cause mortality.

## 4 Discussion

This study, based on a large elderly cohort from Guangzhou (*N* = 424,648), examined the association between NHHR and mortality outcomes among individuals with CKM stages 0–3. A nonlinear association was observed between NHHR and both all-cause and cardio-cerebrovascular disease mortality. Compared with Q3, both Q1 and Q4 were associated with higher mortality risks. RCS analyses suggested a U-shaped pattern, with estimated inflection points at 2.95 and 2.89 for all-cause and cardio-cerebrovascular disease mortality, respectively. Below these thresholds, higher NHHR levels were associated with reduced mortality risk, whereas above them, the risk increased significantly. These findings suggest that the relationship between lipid balance (as reflected by NHHR) and mortality may not be strictly linear, and that intermediate levels may be associated with relatively lower risk in this population.

Our findings are broadly consistent with previous studies reporting associations between NHHR and mortality or metabolic outcomes in different populations. Numerous previous studies across different populations have confirmed that NHHR has good prognostic predictive value. For instance, in hypertensive individuals, elevated NHHR is independently associated with an increased risk of all-cause mortality and cardiovascular mortality, and this association remains significant even after adjusting for traditional risk factors (29). In obese individuals, NHHR exhibits a U-shaped relationship with mortality risk, suggesting that both an excessive burden of atherogenic lipoproteins and abnormally low lipid levels may have adverse effects (30). Furthermore, a study based on the China Health and Retirement Longitudinal Study (CHARLS) also found a significant non-linear (U-shaped) association between NHHR and all-cause mortality in individuals with impaired glucose metabolism (31), further emphasizing the importance of this indicator in metabolically vulnerable populations. Importantly, our study extends the existing literature by demonstrating that these associations persist across different stages of CKM syndrome and exhibit stage-specific heterogeneity. This highlights the potential utility of NHHR not only as a general risk marker but also as a stratification tool in populations with varying cardiometabolic risk profiles.

The U-shaped association observed in this study suggests that both excessively high and low NHHR levels may be detrimental. From a mechanistic perspective, NHHR reflects the balance between atherogenic and antiatherogenic lipoproteins (14, 32), which play central roles in the initiation and progression of atherosclerosis. An elevated NHHR indicates a predominance of atherogenic lipoproteins, particularly non-HDL cholesterol particles (e.g., LDL-C, VLDL, IDL, and remnants), which directly contribute to atherosclerotic plaque formation (33). These lipoproteins can infiltrate the arterial intima, undergo oxidative modification, and promote foam cell formation, while also activating inflammatory pathways such as the NLRP3 inflammasome and TLR4/NF-kB axis, thereby accelerating vascular inflammation and plaque instability (33–35). In addition, cumulative exposure to elevated non-HDL cholesterol has been strongly linked to increased cardiovascular risk and mortality (36), supporting the biological plausibility of the observed association between high NHHR and adverse outcomes. In contrast, a low NHHR does not necessarily indicate a favorable lipid profile but may instead reflect underlying pathological conditions. First, reduced NHHR levels are often driven by decreased TC or non-HDL cholesterol, both of which have been associated with malnutrition, frailty, and chronic disease, particularly in older populations (37–39). Second, in individuals with CKM syndrome, advanced disease stages are frequently accompanied by profound metabolic dysregulation, including mitochondrial dysfunction, insulin resistance, and impaired energy metabolism (2, 40). These alterations are often associated with catabolic states and systemic inflammation, which may contribute to lower lipid levels. Indeed, accumulating evidence suggests that higher CKM stages are strongly associated with increased risks of all-cause and cardiovascular mortality (41). Finally, although HDL-C is traditionally considered protective (38), its functionality may be impaired under conditions such as aging, chronic inflammation, and CKM syndrome, resulting in reduced cholesterol efflux capacity and even pro-inflammatory effects (42, 43). Therefore, in this context, a low NHHR may serve as a marker of diminished physiological reserve rather than a protective factor, thereby contributing to an increased risk of mortality. Taken together, these findings provide a plausible biological explanation for the observed U-shaped association between NHHR and mortality, where both excessive atherogenic burden and impaired metabolic or nutritional status may contribute to increased risk.

Subgroup and interaction analyses demonstrated that sex, smoking status, age, and CKM stage significantly modified the association between NHHR and mortality, indicating substantial heterogeneity across populations. Specifically, men exhibited a higher mortality risk than women in response to elevated NHHR levels, suggesting greater susceptibility to metabolic disturbances. For instance, Zhou et al. reported that men faced a higher mortality risk with increasing uric acid-to-HDL-C ratio, suggesting that factors such as hormonal environment, body composition, and metabolic reserves may render men more vulnerable to metabolic disturbances (44). Similarly, Yang et al. found that men with early-onset cardiovascular disease were more susceptible to adverse outcomes triggered by metabolic disorders (45). Smoking status also significantly interacted with NHHR in relation to cardio-cerebrovascular disease mortality. Previous studies have shown that smoking, as a major exogenous risk factor for cardiovascular disease, accelerates atherosclerosis through mechanisms including oxidative stress, chronic inflammation, endothelial dysfunction, and dyslipidemia (46). Therefore, smoking, in combination with the intrinsic lipid dysregulation reflected by high NHHR, may exert a synergistic effect, jointly exacerbating atherosclerosis and amplifying mortality risk. Among older individuals, particularly the elderly, the increase in mortality risk associated with high NHHR was more pronounced, which may be attributed to age-related declines in physiological reserve, increased arterial stiffness, and a pro-inflammatory milieu (47–49). Similarly, the adverse impact of NHHR was most evident in advanced CKM stage 3; this is likely driven by the cumulative burden of metabolic, cardiovascular, and renal dysfunction, which synergistically exacerbate disease progression and vulnerability to adverse outcomes (50). Stage-stratified restricted cubic spline analyses further showed that although the U-shaped association between NHHR and mortality persisted across CKM stages, the inflection points shifted with disease progression. In CKM stage 0, the inflection point occurred at a relatively lower NHHR level, suggesting that metabolically healthier individuals may be more sensitive to early lipid imbalance. In contrast, higher thresholds observed in more advanced stages may reflect disease-related metabolic alterations or reduced sensitivity to lipid imbalance (51). Furthermore, the observation that the lowest NHHR quartile (Q1) was associated with higher mortality risk than Q3 across multiple subgroups reinforces the concept that a moderate lipid balance may be optimal. Previous studies have demonstrated a U-shaped relationship between NHHR and mortality, with the highest risk observed in Q1 (29). This paradoxical finding may be partially explained by dysfunctional HDL or reduced non-HDL cholesterol levels secondary to underlying chronic diseases (42, 52), highlighting the limitations of relying on single lipid markers for risk assessment. Collectively, these findings underscore the importance of integrating individual characteristics—such as age, sex, smoking behavior, and CKM stage—when interpreting the prognostic value of NHHR to enable more precise risk stratification and targeted interventions.

Mediation analysis suggested that ePWV may partially explain the association between NHHR and cardio-cerebrovascular disease mortality, accounting for approximately 40.16% of the association. This suggests that a substantial portion of the detrimental impact of NHHR on the cardio-cerebrovascular systems is mediated by promoting arterial stiffness. The underlying biological pathway can be explained as follows: both excessively high and low NHHR levels indicate dyslipidemia. It can induce vascular endothelial inflammation, oxidative stress, and dysfunction, which in turn leads to structural changes in the vascular wall and increased stiffness, ultimately manifesting as elevated ePWV (53). High ePWV, indicative of increased arterial rigidity, raises cardiac afterload, widens pulse pressure, and impairs organ perfusion (49). This hemodynamic burden can ultimately induce heart failure and increase the risk of cardio-cerebrovascular death (54, 55). However, given the observational design and the use of derived ePWV estimates, these results should be interpreted as exploratory rather than causal evidence. Notably, this mediating effect was not observed for all-cause mortality, suggesting that NHHR influences overall health through multiple pathways beyond the atherosclerotic-stiffness-cardiovascular axis. NHHR may also contribute to all-cause mortality by promoting non-cardiovascular conditions, such as insulin resistance (56), non-alcoholic fatty liver disease (NAFLD) (57), and chronic kidney disease (58). These diverse pathways collectively form the complex network through which NHHR affects health. For instance, prior research suggests that the association between NHHR and all-cause mortality in individuals with Non-Hyperhomocysteinemia (NHHcy) is mediated by aspartate aminotransferase (59). Future studies are warranted to elucidate these potential pathways further.

This study has several notable strengths. First, it evaluates the association between NHHR and mortality risks in a large cohort of older adults with CKM syndrome stages 0–3, providing evidence within a stage-based cardiometabolic framework that has been less frequently addressed in previous studies. Second, we employed a comprehensive and rigorous analytical strategy, including Cox proportional hazards models, restricted cubic spline analyses, threshold effect modeling, subgroup and interaction analyses, mediation analysis, and multiple sensitivity analyses. This multifaceted approach enhances the robustness, reliability, and interpretability of our findings. Third, subgroup and exploratory mediation analyses were conducted to examine potential heterogeneity and underlying pathways. These analyses suggest that the association between NHHR and mortality may vary across population subgroups. That arterial stiffness may be related to the observed association with cardio-cerebrovascular disease mortality.

Although this study has several strengths, several limitations should be acknowledged. First, the study population consisted of older adults aged ≥65 years from southern China. Therefore, the generalizability of the findings to younger populations or to individuals from different ethnic and geographic backgrounds may be limited, and external validation is warranted. Second, NHHR was assessed only at baseline, and changes over time or cumulative exposure were not captured. This may lead to exposure misclassification and limit the ability to reflect long-term lipid dynamics. Third, although multiple demographic, lifestyle, and clinical covariates were adjusted for, some variables were based on self-reported information and may be subject to recall bias. Fourth, due to data availability, several clinically relevant variables, including inflammatory markers, nutritional indicators, frailty-related measures, and more granular cardiovascular and renal disease severity indices, were not available. Residual confounding, therefore, cannot be excluded. Fifth, ePWV is a derived measure based on age and blood pressure. Although it is widely used as a surrogate marker of arterial stiffness, potential conceptual overlap and residual collinearity with exposure variables may exist. Accordingly, the mediation results should be interpreted as exploratory rather than causal evidence. In addition, the identification of inflection points and threshold effects was based on data-driven modeling approaches. While these analyses help describe potential nonlinear relationships, the estimated values should not be interpreted as precise or clinically actionable cut-points, and their uncertainty may not be fully captured. Moreover, renal function was assessed using the standard eGFR threshold (≥60 mL/min/1.73 m^2^) without age-adjusted criteria. Given the physiological decline in renal function with aging, this approach may introduce potential misclassification in this elderly population. Finally, the observational design of this study precludes causal inference. Further validation in independent cohorts and prospective or interventional studies is warranted before any clinical translation can be considered.

## 5 Conclusion

This large cohort study of elderly individuals with CKM stages 0–3 found that NHHR was associated with both all-cause and cardio-cerebrovascular disease mortality, with a consistent U-shaped pattern observed across analyses. The lowest risk was observed at intermediate NHHR levels, while both lower and higher values were associated with increased mortality risk. These associations remained stable across multiple sensitivity analyses and CKM stage stratifications. Overall, these findings suggest that NHHR may be associated with mortality risk in this population and may serve as an exploratory biomarker of cardiometabolic risk.

## Data Availability

The data analyzed in this study is subject to the following licenses/restrictions: due to the privacy concerns of individuals who participated in the research, the data underlying this paper cannot be publicly shared. The data will be shared on a reasonable request to the corresponding author. Requests to access these datasets should be directed to ZZ, zhangzhb26@mail2.sysu.edu.cn.

## Glossary

GOLD-Health: Guangzhou older longitudinal dynamic health
CKM syndrome: Cardiovascular-kidney-metabolic syndrome
NHHR: Non-high-density lipoprotein cholesterol to high-density lipoprotein cholesterol ratio
ePWV: Estimated pulse wave velocity
AHA: American heart association
CVD: Cardiovascular disease
CKD: Chronic kidney disease
LDL-C: Low-density lipoprotein cholesterol
HDL-C: High-density lipoprotein cholesterol
TC: Total cholesterol
eGFR: Estimated Glomerular Filtration Rate
Scr: Serum creatinine
KDIGO: Kidney disease improving global outcomes
ICD-10: International classification of diseases, tenth revision
VIF: Variance inflation factor
PA: Physical activity
BMI: Body mass index
SD: Standard deviation
SMD: Standardized mean difference
IQR: Interquartile range
RCS: Restricted cubic spline
HR: Hazard ratio
95% CI: 95% confidence interval
IPCW: inverse probability of censoring weighting
WHO: World Health Organization
MBP: Mean blood pressure
SBP: Systolic blood pressure
DBP: Diastolic blood pressure
WC: Waist circumference
FBG: Fasting blood glucose
TG: Triglyceride
CHARLS: China health and retirement longitudinal study
NAFLD: Non-alcoholic fatty liver disease
NHHcy: Non-hyperhomocysteinemia
MetS: Metabolic syndrome
HF: Heart failure
