# Supplementary material for: Association of non-high-density lipoprotein cholesterol to high-density lipoprotein cholesterol ratio with all-cause mortality and cardio-cerebrovascular disease mortality in elderly patients with cardiovascular-kidney-metabolic syndrome stages 0–3: a cohort study
Source: Front Nutr. 2026 May 29;13:1761136. doi: 10.3389/fnut.2026.1761136 (PMC13259738; doi:10.3389/fnut.2026.1761136)
Supplement: Supplementary file 1 [file Table_1.docx]

**Association of non-high-density lipoprotein cholesterol to high-density lipoprotein cholesterol ratio with all-cause mortality and cardio-cerebrovascular disease mortality in elderly patients with cardiovascular-kidney-metabolic syndrome stages 0-****3:** **A cohort study**

**Supplementary material**

**Table S1** Framingham risk score for men.

**Table S2** CVD Risk for Men.

**Table S3** Framingham risk score for women.

Table S4 CVD Risk for Women.

**Table S5** Definitions of CKM Syndrome Stage.

**Table S6** Specific definitions of various diseases.

**Table S7** Assessment of multicollinearity among independent variables.

**Table S8** Baseline characteristics of included and excluded participants to assess potential selection bias.

**Table S9** Subgroup analysis of the association between NHHR and all-cause mortality in the elderly population with CKM stages 0-3.

**Table S10** Subgroup analysis of the association between NHHR and cardio-cerebrovascular disease mortality in the elderly population with CKM stages 0-3.

**Table S11** Sensitivity analysis: Associations of NHHR quartiles with mortality risk: results from complete-case and IPCW-weighted Cox models.

**Table S12** Sensitivity analysis: Associations of NHHR quartiles with mortality using Q1 as the reference group.

**Table S13** Sensitivity analysis: Associations of NHHR quartiles with mortality after excluding participants with self-reported cancer at baseline (N=418697).

**Table S14** Sensitivity analysis: Associations of NHHR quartiles with mortality after excluding participants from the first 2 years of follow-up (N=412259).

**Table S15** Sensitivity analysis: Associations of NHHR quartiles with mortality after additional adjustment for lipid-lowering medication.

**Table S16** Sensitivity analysis: Associations of NHHR quartiles with mortality after adjustment using WHO BMI classifications.

**Figure S1** Scaled Schoenfeld residual plot for NHHR quantiles in the mortality model.

**Figure S2** Restricted cubic spline analyses of the associations between NHHR and mortality across CKM stages 0-3.

**Table S1 Framingham risk score for men.**

| **Points** | **Age, years** | **HDL-C** | **TC** | **SBP Not Treated** | **SBP Treated** | **Smoker** | **Diabetic** |
| --- | --- | --- | --- | --- | --- | --- | --- |
| -2 |  | ≥60 |  | <120 |  |  |  |
| -1 |  | [50–59] |  |  |  |  |  |
| 0 | 30–34 | [45–49] | <160 | [120–129] | <120 | No | No |
| 1 |  | [35–44] | [160–199] | [130–139] |  |  |  |
| 2 | 35–39 | <35 | [200–239] | [140–159] | [120–129] |  |  |
| 3 |  |  | [240–279] | ≥160 | [130–139] |  | Yes |
| 4 |  |  | ≥280 |  | [140–159] | Yes |  |
| 5 | 40–44 |  |  |  | ≥160+ |  |  |
| 6 | 45–49 |  |  |  |  |  |  |
| 7 |  |  |  |  |  |  |  |
| 8 | 50–54 |  |  |  |  |  |  |
| 9 |  |  |  |  |  |  |  |
| 10 | 55–59 |  |  |  |  |  |  |
| 11 | 60–64 |  |  |  |  |  |  |
| 12 | 65–69 |  |  |  |  |  |  |
| 13 |  |  |  |  |  |  |  |
| 14 | 70–74 |  |  |  |  |  |  |
| 15 | 75+ |  |  |  |  |  |  |

Note: HDL-C: high-density lipoprotein cholesterol; TC: total cholesterol; SBP: systolic blood pressure.

**Table S2 CVD Risk for Men.**

| **Points** | **Risk, %** | **Points** | **Risk, %** | **Points** | **Risk, %** |
| --- | --- | --- | --- | --- | --- |
| -3 or less | <1 | 5 | 3.9 | 13 | 15.6 |
| -2 | 1.1 | 6 | 4.7 | 14 | 18.4 |
| -1 | 1.4 | 7 | 5.6 | 15 | 21.6 |
| 0 | 1.6 | 8 | 6.7 | 16 | 25.3 |
| 1 | 1.9 | 9 | 7.9 | 17 | 29.4 |
| 2 | 2.3 | 10 | 9.4 | 18+ | >30 |
| 3 | 2.8 | 11 | 11.2 |  |  |
| 4 | 3.3 | 12 | 13.2 |  |  |

**Table S3 Framingham risk score for women.**

| **Points** | **Age, years** | **HDL-C** | **TC** | **SBP Not Treated** | **SBP Treated** | **Smoker** | **Diabetic** |
| --- | --- | --- | --- | --- | --- | --- | --- |
| -3 |  |  |  | <120 |  |  |  |
| -2 |  | ≥60 |  |  |  |  |  |
| -1 |  | [50–59] |  |  | <120 |  |  |
| 0 | 30–34 | [45–49] | <160 | [120–129] |  | No | No |
| 1 |  | [35–44] | [160–199] | [130–139] |  |  |  |
| 2 | 35–39 | <35 |  | [140–149] | [120–129] |  |  |
| 3 |  |  | [200–239] |  | [130–139] | Yes |  |
| 4 | 40–44 |  | [240–279] | [150–159] |  |  | Yes |
| 5 | 45–49 |  | ≥280 | ≥160 | [140–149] |  |  |
| 6 |  |  |  |  | [150–159] |  |  |
| 7 | 50–54 |  |  |  | ≥160 |  |  |
| 8 | 55–59 |  |  |  |  |  |  |
| 9 | 60–64 |  |  |  |  |  |  |
| 10 | 65–69 |  |  |  |  |  |  |
| 11 | 70–74 |  |  |  |  |  |  |
| 12 | 75+ |  |  |  |  |  |  |

Note: HDL-C: high-density lipoprotein cholesterol; TC: total cholesterol; SBP: systolic blood pressure.

**Table S4 CVD Risk for Women.**

| **Points** | **Risk, %** | **Points** | **Risk, %** | **Points** | **Risk, %** |
| --- | --- | --- | --- | --- | --- |
| -2 or less | <1 | 6 | 3.3 | 14 | 11.7 |
| -1 | 1.0 | 7 | 3.9 | 15 | 13.7 |
| 0 | 1.2 | 8 | 4.5 | 16 | 15.9 |
| 1 | 1.5 | 9 | 5.3 | 17 | 18.5 |
| 2 | 1.7 | 10 | 6.3 | 18 | 21.5 |
| 3 | 2.0 | 11 | 7.3 | 19 | 24.8 |
| 4 | 2.4 | 12 | 8.6 | 20 | 28.5 |
| 5 | 2.8 | 13 | 10.0 | 21+ | >30 |

**Table S5 Definitions of CKM Syndrome Stage.**

| **CKM stage** | **Threshold for CKM conditions** |
| --- | --- |
| Stage 0: No CKM risk factors | Individuals with normal BMI and waist circumference, normoglycemia, normotension, a normal lipid profile, and no evidence of CKD or subclinical or clinical CVD |
| Stage 1: Excess or  dysfunctional adiposity | Individuals with overweight/obesity, abdominal obesity, or dysfunctional adipose tissue, without the presence of other metabolic risk factors or CKD |
| Stage 2: Metabolic risk factors and CKD | Individuals with metabolic risk factors (hypertriglyceridemia, hypertension, MetS, diabetes), or CKD |
| Stage 3: Subclinical CVD in CKM | Very high-risk CKD (stage G4 or G5 CKD) or high predicted 10-year CVD risk |
| Stage 4: Clinical CVD in CKM | Clinical CVD (coronary heart disease, HF, stroke, peripheral artery disease, atrial fibrillation) among individuals with excess/dysfunctional adiposity, other CKM risk factors, or CKD. |

Note: CKM stage: Cardiovascular-kidney-metabolic syndrome stage, CKD: Chronic kidney disease, CVD: Cardiovascular Disease, MetS: Metabolic syndrome, HF: heart failure.

**Table S6 Specific definitions of various diseases.**

| **Diseases** | **Definition** |
| --- | --- |
| Overweight/obesity | BMI ≥ 23 kg/m^2^ |
| Abdominal obesity | Waist circumference ≥ 80/90 cm in female/male |
| Prediabetes | Fasting blood glucose ≥ 100-124 mg/dL and without self-reported diagnosis of diabetes, use of insulin, or oral hypoglycemic agents |
| Diabetes | Fasting blood glucose ≥ 125 mg/dL or self-reported diagnosis of diabetes, use of insulin, or oral hypoglycemic agents |
| Hypertension | SBP ≥130 mmHg or DBP ≥ 80 mmHg or self-reported diagnosis of hypertension or use of antihypertensive medications |
| Chronic kidney disease | Either of the two criteria is met:  ① Self-reported history of chronic kidney disease.  ② eGFR < 60 ml/min/1.73m^2^. |
| Hypertriglyceridemia | Triglycerides ≥ 135 mg/dL |
| Metabolic syndrome | Meet any three or more of the five:  ① Waist circumference ≥ 80/90 cm in females/males.  ② HDL cholesterol < 50/40 mg/dL in females/males.  ③ Triglycerides ≥ 150 mg/dL.  ④ Elevated blood pressure (SBP ≥ 130 mmHg or DBP ≥ 80 mmHg and/or use of antihypertensive medications).  ⑤ Fasting blood glucose ≥ 100 mg/dL. |

**Table S7 Assessment of multicollinearity among independent variables**

| **Variables** | **GVIF** | **Df** | **GVIF^1/2Df^** |
| --- | --- | --- | --- |
| NHHR | 1.05476233 | 1 | 1.027016227 |
| Sex | 1.414435974 | 1 | 1.189300624 |
| Age groups | 1.147944618 | 2 | 1.035095056 |
| Marital status | 1.087772999 | 1 | 1.042963565 |
| Education level | 1.134064958 | 4 | 1.015850366 |
| Smoking status | 1.435835997 | 1 | 1.198263743 |
| Drinking status | 1.165114741 | 1 | 1.079404809 |
| PA | 1.056169679 | 3 | 1.009149748 |
| BMI | 1.101172062 | 2 | 1.024386381 |
| Antihypertensive medication | 1.080440964 | 1 | 1.039442622 |
| Antidiabetic medication | 1.038667422 | 1 | 1.019150343 |

Note: BMI: Body mass index, PA: Physical Activity.

**Table S8 Baseline characteristics of included and excluded participants to assess potential selection bias.**

| **Variables** | **Excluded (N=49897)** | **Included (N=42468)** | ***P* value** | **SMD** |
| --- | --- | --- | --- | --- |
| **Education level (%)** |  |  | <0.001 | 0.142 |
| Illiterate | 6052 (12.9) | 45296 (10.7) |  |  |
| Primary school | 17265 (36.7) | 135660 (31.9) |  |  |
| Middle school | 11388 (24.2) | 112906 (26.6) |  |  |
| High school | 3539 (7.5) | 38324 (9.0) |  |  |
| University or higher | 8819 (18.7) | 92462 (21.8) |  |  |
| **Drinking status (%)** |  |  | 0.007 | 0.014 |
| No | 41331 (94.5) | 400132 (94.2) |  |  |
| Yes | 2386 (5.5) | 24516 (5.8) |  |  |
| **PA (%)** |  |  | <0.001 | 0.065 |
| No exercise | 12299 (26.2) | 118870 (28.0) |  |  |
| Occasional | 3632 (7.7) | 27182 (6.4) |  |  |
| Weekly or more | 11246 (24.0) | 104704 (24.7) |  |  |
| Daily | 19701 (42.0) | 173892 (40.9) |  |  |
| **Height**  **(mean ± SD)** | 1.58 ± 0.33 | 1.57 ± 0.08 | <0.001 | 0.051 |
| **Weight**  **(mean ± SD)** | 60.25 ± 11.56 | 58.09 ± 9.91 | <0.001 | 0.201 |
| **WC**  **(mean ± SD)** | 85.72 ± 9.81 | 83.57 ± 8.62 | <0.001 | 0.233 |
| **FBG**  **(mean ± SD)** | 105.01 ± 24.51 | 102.22 ± 21.86 | <0.001 | 0.12 |
| **Scr (mean ± SD)** | 0.94 ± 0.35 | 0.93 ± 0.32 | <0.001 | 0.052 |
| **TC (mean ± SD)** | 219.15 ± 49.75 | 208.94 ± 40.96 | <0.001 | 0.224 |
| **TG (mean ± SD)** | 155.13 ± 79.60 | 139.58 ± 69.83 | <0.001 | 0.208 |
| **HDL_C**  **(mean ± SD)** | 53.66 ± 15.69 | 56.76 ± 14.39 | <0.001 | 0.206 |
| **LDL_C**  **(mean ± SD)** | 124.13 ± 40.60 | 126.02 ± 34.81 | <0.001 | 0.05 |
| **NHHR**  **(mean ± SD)** | 3.10 ± 1.61 | 2.86 ± 1.04 | <0.001 | 0.173 |

Note: SMD: Standardized mean difference, PA: Physical activity, SD: standard deviation, WC: Waist circumference, FBG: Fasting blood glucose, Scr: Serum creatinine, TC: Total cholesterol, TG: Triglyceride, HDL-C: High-density lipoprotein cholesterol, LDL-C: Low-density lipoprotein cholesterol.

**Table S9 Subgroup analysis of the association between NHHR and all-cause mortality in the elderly population with CKM stages 0-3.**

| **Variable** | **Count (%)** | **Levels** | **HR (95% CI)** | ***P* value** | ***P* for interaction** |
| --- | --- | --- | --- | --- | --- |
| Overall | 424648 (100) | Q3 |  |  |  |
|  |  | Q1 | 1.13 (1.10-1.16) | <0.001 |  |
|  |  | Q2 | 1.02 (0.99-1.05) | 0.246 |  |
|  |  | Q4 | 1.07 (1.04-1.10) | <0.001 |  |
| Sex |  |  |  |  | 0.021 |
| Male | 169486 (39.9) | Q3 |  |  |  |
|  |  | Q1 | 1.14 (1.10-1.19) | <0.001 |  |
|  |  | Q2 | 1.01 (0.97-1.05) | 0.553 |  |
|  |  | Q4 | 1.09 (1.05-1.13) | <0.001 |  |
| Female | 255162 (60.1) | Q3 |  |  |  |
|  |  | Q1 | 1.13 (1.08-1.17) | <0.001 |  |
|  |  | Q2 | 1.03 (0.99-1.07) | 0.201 |  |
|  |  | Q4 | 1.05 (1.01-1.09) | 0.026 |  |
| Age groups |  |  |  |  | 0.013 |
| 65-69 | 185524 (43.7) | Q3 |  |  |  |
|  |  | Q1 | 1.15 (1.07-1.23) | <0.001 |  |
|  |  | Q2 | 1.03 (0.96-1.11) | 0.389 |  |
|  |  | Q4 | 1.13 (1.06-1.21) | <0.001 |  |
| 70-79 | 172520 (40.6) | Q3 |  |  |  |
|  |  | Q1 | 1.13 (1.08-1.18) | <0.001 |  |
|  |  | Q2 | 1.02 (0.97-1.07) | 0.463 |  |
|  |  | Q4 | 1.09 (1.04-1.14) | <0.001 |  |
| ≥80 | 66604 (15.7) | Q3 |  |  |  |
|  |  | Q1 | 1.12 (1.08-1.17) | <0.001 |  |
|  |  | Q2 | 1.01 (0.97-1.05) | 0.527 |  |
|  |  | Q4 | 1.03 (0.99-1.07) | 0.195 |  |
| Smoking status |  |  |  |  | <0.001 |
| No | 361339 (85.1) | Q3 |  |  |  |
|  |  | Q1 | 1.15 (1.11-1.18) | <0.001 |  |
|  |  | Q2 | 1.02 (0.99-1.05) | 0.273 |  |
|  |  | Q4 | 1.06 (1.03-1.10) | <0.001 |  |
| Yes | 63309 (14.9) | Q3 |  |  |  |
|  |  | Q1 | 1.06 (0.99-1.13) | 0.089 |  |
|  |  | Q2 | 1.02 (0.96-1.09) | 0.565 |  |
|  |  | Q4 | 1.09 (1.03-1.17) | 0.005 |  |

(Continued)

**Table S9 (Continued)**

| **Variable** | **Count (%)** | **Levels** | **HR (95% CI)** | ***P* value** | ***P* for interaction** |
| --- | --- | --- | --- | --- | --- |
| Drinking status |  |  |  |  | 0.21 |
| No | 400132 (94.2) | Q3 |  |  |  |
|  |  | Q1 | 1.13 (1.10-1.16) | <0.001 |  |
|  |  | Q2 | 1.01 (0.98-1.04) | 0.39 |  |
|  |  | Q4 | 1.07 (1.04-1.10) | <0.001 |  |
| Yes | 24516 (5.8) | Q3 |  |  |  |
|  |  | Q1 | 1.16 (1.03-1.30) | 0.013 |  |
|  |  | Q2 | 1.09 (0.97-1.23) | 0.146 |  |
|  |  | Q4 | 1.16 (1.03-1.31) | 0.015 |  |
| CKM stages |  |  |  |  | <0.001 |
| 0 | 22271 (5.2) | Q3 |  |  |  |
|  |  | Q1 | 0.98 (0.84-1.15) | 0.833 |  |
|  |  | Q2 | 0.95 (0.80-1.12) | 0.511 |  |
|  |  | Q4 | 1.05 (0.82-1.34) | 0.723 |  |
| 1 | 29037 (6.8) | Q3 |  |  |  |
|  |  | Q1 | 1.20 (1.03-1.38) | 0.015 |  |
|  |  | Q2 | 1.08 (0.93-1.26) | 0.328 |  |
|  |  | Q4 | 1.14 (0.91-1.43) | 0.24 |  |
| 2 | 150429 (35.4) | Q3 |  |  |  |
|  |  | Q1 | 1.19 (1.13-1.25) | <0.001 |  |
|  |  | Q2 | 1.03 (0.97-1.09) | 0.302 |  |
|  |  | Q4 | 1.03 (0.96-1.10) | 0.408 |  |
| 3 | 222911 (52.5) | Q3 |  |  |  |
|  |  | Q1 | 1.13 (1.10-1.17) | <0.001 |  |
|  |  | Q2 | 1.03 (1.00-1.06) | 0.093 |  |
|  |  | Q4 | 1.06 (1.03-1.10) | <0.001 |  |

Note: HR: hazard ratio, 95 % CI: 95% confidence interval, BMI: Body mass index, CKM stages: Cardiovascular-kidney-metabolic syndrome stages.

**Table S10 Subgroup analysis of the association between NHHR and cardio-cerebrovascular disease mortality in the elderly population with CKM stages 0-3.**

| **Variable** | **Count (%)** | **Levels** | **HR (95% CI)** | ***P* value** | ***P* for interaction** |
| --- | --- | --- | --- | --- | --- |
| Overall | 424648 (100) | Q3 |  |  |  |
|  |  | Q1 | 1.09 (1.05-1.13) | <0.001 |  |
|  |  | Q2 | 0.99 (0.95-1.03) | 0.577 |  |
|  |  | Q4 | 1.11 (1.07-1.16) | <0.001 |  |
| Sex |  |  |  |  | <0.001 |
| Male | 169486 (39.9) | Q3 |  |  |  |
|  |  | Q1 | 1.09 (1.02-1.15) | 0.007 |  |
|  |  | Q2 | 0.97 (0.91-1.03) | 0.314 |  |
|  |  | Q4 | 1.13 (1.06-1.20) | <0.001 |  |
| Female | 255162 (60.1) | Q3 |  |  |  |
|  |  | Q1 | 1.10 (1.04-1.16) | 0.001 |  |
|  |  | Q2 | 1.01 (0.96-1.07) | 0.666 |  |
|  |  | Q4 | 1.08 (1.02-1.14) | 0.007 |  |
| Age groups |  |  |  |  | <0.001 |
| 65-69 | 185524 (43.7) | Q3 |  |  |  |
|  |  | Q1 | 1.14 (1.01-1.29) | 0.035 |  |
|  |  | Q2 | 1.02 (0.91-1.16) | 0.701 |  |
|  |  | Q4 | 1.25 (1.11-1.40) | <0.001 |  |
| 70-79 | 172520 (40.6) | Q3 |  |  |  |
|  |  | Q1 | 1.08 (1.00-1.15) | 0.041 |  |
|  |  | Q2 | 0.99 (0.92-1.07) | 0.826 |  |
|  |  | Q4 | 1.15 (1.07-1.23) | <0.001 |  |
| ≥80 | 66604 (15.7) | Q3 |  |  |  |
|  |  | Q1 | 1.08 (1.02-1.14) | 0.004 |  |
|  |  | Q2 | 0.98 (0.93-1.04) | 0.514 |  |
|  |  | Q4 | 1.05 (0.99-1.11) | 0.131 |  |
| Smoking status |  |  |  |  | <0.001 |
| No | 361339 (85.1) | Q3 |  |  |  |
|  |  | Q1 | 1.10 (1.05-1.15) | <0.001 |  |
|  |  | Q2 | 0.99 (0.95-1.04) | 0.751 |  |
|  |  | Q4 | 1.09 (1.04-1.14) | <0.001 |  |
| Yes | 63309 (14.9) | Q3 |  |  |  |
|  |  | Q1 | 1.01 (0.91-1.12) | 0.819 |  |
|  |  | Q2 | 0.97 (0.87-1.08) | 0.561 |  |
|  |  | Q4 | 1.18 (1.07-1.30) | 0.001 |  |

(Continued)

**Table S10 (Continued)**

| **Variable** | **Count (%)** | **Levels** | **HR (95% CI)** | ***P* value** | ***P* for interaction** |
| --- | --- | --- | --- | --- | --- |
| Drinking status |  |  |  |  | 0.068 |
| No | 400132 (94.2) | Q3 |  |  |  |
|  |  | Q1 | 1.09 (1.05-1.14) | <0.001 |  |
|  |  | Q2 | 0.99 (0.95-1.03) | 0.599 |  |
|  |  | Q4 | 1.10 (1.06-1.15) | <0.001 |  |
| Yes | 24516 (5.8) | Q3 |  |  |  |
|  |  | Q1 | 1.05 (0.87-1.27) | 0.635 |  |
|  |  | Q2 | 0.98 (0.80-1.19) | 0.821 |  |
|  |  | Q4 | 1.29 (1.06-1.57) | 0.01 |  |
| CKM stages |  |  |  |  | <0.001 |
| 0 | 22271 (5.2) | Q3 |  |  |  |
|  |  | Q1 | 0.80 (0.62-1.03) | 0.09 |  |
|  |  | Q2 | 0.85 (0.65-1.12) | 0.255 |  |
|  |  | Q4 | 0.89 (0.59-1.36) | 0.605 |  |
| 1 | 29037 (6.8) | Q3 |  |  |  |
|  |  | Q1 | 0.98 (0.78-1.24) | 0.897 |  |
|  |  | Q2 | 0.90 (0.70-1.16) | 0.432 |  |
|  |  | Q4 | 0.97 (0.67-1.41) | 0.87 |  |
| 2 | 150429 (35.4) | Q3 |  |  |  |
|  |  | Q1 | 1.22 (1.13-1.32) | <0.001 |  |
|  |  | Q2 | 1.06 (0.97-1.15) | 0.183 |  |
|  |  | Q4 | 1.10 (0.99-1.21) | 0.068 |  |
| 3 | 222911 (52.5) | Q3 |  |  |  |
|  |  | Q1 | 1.10 (1.04-1.16) | <0.001 |  |
|  |  | Q2 | 1.00 (0.95-1.05) | 0.996 |  |
|  |  | Q4 | 1.08 (1.04-1.14) | 0.001 |  |

Note: HR: hazard ratio, 95 % CI: 95% confidence interval, BMI: Body mass index, CKM stages: Cardiovascular-kidney-metabolic syndrome stages.

**Table S11 Sensitivity analysis: Associations of NHHR quartiles with mortality risk: results from complete-case and IPCW-weighted Cox models**

| **Variables** | **Model 1,**  **HR (95% CI) *P* value** | **Model 2,**  **HR (95 % CI) *P* value** |
| --- | --- | --- |
| **All-cause mortality** | | |
| Q3 | Ref | Ref |
| Q1 | 1.13 (1.10–1.16) <0.001 | 1.14 (1.11–1.17) <0.001 |
| Q2 | 1.01 (0.99–1.04) 0.300 | 1.02 (0.99–1.04) 0.165 |
| Q4 | 1.08 (1.05–1.11) <0.001 | 1.08 (1.05–1.11) <0.001 |
| **Cardio-cerebrovascular disease mortality** | | |
| Q3 | Ref | Ref |
| Q1 | 1.08 (1.04–1.13) <0.001 | 1.10 (1.06–1.14) <0.001 |
| Q2 | 0.98 (0.94–1.02) 0.392 | 0.99 (0.95–1.02) 0.465 |
| Q4 | 1.11 (1.07–1.16) <0.001 | 1.11 (1.07–1.15) <0.001 |

Note: HR: hazard ratio, 95% CI: 95% confidence interval, IPCW: inverse probability of censoring weighting.

Model 1 was based on complete-case analysis, and Model 2 was derived from IPCW analysis. All models were adjusted for sex, age groups, marital status, BMI, education level, smoking status, drinking status, PA, antihypertensive medication, and antidiabetic medication.

BMI: Body mass index, PA: Physical activity

**Table S12 Sensitivity analysis: Associations of NHHR quartiles with mortality using Q1 as the reference group.**

| **Variables** | **Number of deaths** | **Model 1,**  **HR (95% CI) *P* value** | **Model 2,**  **HR (95 % CI) *P* value** | **Model 3,**  **HR (95% CI) *P* value** |
| --- | --- | --- | --- | --- |
| **All-cause mortality** | | | | |
| Q1 | 13129 | Ref | Ref | Ref |
| Q2 | 10543 | 0.79 (0.77-0.81) <0.001 | 0.86 (0.84-0.88) <0.001 | 0.90 (0.87-0.92) <0.001 |
| Q3 | 9600 | 0.71 (0.70-0.73) <0.001 | 0.82 (0.80-0.84) <0.001 | 0.88 (0.86-0.91) <0.001 |
| Q4 | 10185 | 0.76 (0.74-0.78) <0.001 | 0.87 (0.84-0.89) <0.001 | 0.95 (0.92-0.97) <0.001 |
| **Cardio-cerebrovascular disease mortality** | | | | |
| Q1 | 5762 | Ref | Ref | Ref |
| Q2 | 4669 | 0.80 (0.77-0.83) <0.001 | 0.88 (0.85-0.91) <0.001 | 0.91 (0.87-0.94) <0.001 |
| Q3 | 4363 | 0.74 (0.71-0.77) <0.001 | 0.86 (0.83-0.90) <0.001 | 0.92 (0.88-0.96) <0.001 |
| Q4 | 4796 | 0.81 (0.78-0.85) <0.001 | 0.95 (0.92-0.99) <0.001 | 1.02 (0.98-1.06) <0.001 |

Note: HR: hazard ratio, 95%CI: 95% confidence interval,

Model 1: Crude model. Model 2: Adjusted for sex and age groups. Model 3: Adjusted for sex, age groups, marital status, BMI, education level, smoking status, drinking status, PA, antihypertensive medication, and antidiabetic medication.

BMI: Body mass index, PA: Physical activity.

**Table S13 Sensitivity analysis: Associations of NHHR quartiles with mortality after excluding participants with self-reported cancer at baseline (N = 418697).**

| **Variables** | **Number of deaths** | **Model 1,**  **HR (95% CI) *P* value** | **Model 2,**  **HR (95 % CI) *P* value** | **Model 3,**  **HR (95% CI) *P* value** |
| --- | --- | --- | --- | --- |
| **All-cause mortality** | | | | |
| Q3 | 9230 | Ref | Ref | Ref |
| Q1 | 12708 | 1.41 (1.37-1.45) <0.001 | 1.23 (1.20-1.26) <0.001 | 1.14 (1.11-1.17) <0.001 |
| Q2 | 10175 | 1.11 (1.08-1.14) <0.001 | 1.05 (1.03-1.08) <0.001 | 1.02 (0.99-1.05) 0.197 |
| Q4 | 9802 | 1.06 (1.03-1.09) <0.001 | 1.06 (1.03-1.09) <0.001 | 1.07 (1.04-1.10) <0.001 |
| **Cardio-cerebrovascular disease mortality** | | | | |
| Q3 | 4300 | Ref | Ref | Ref |
| Q1 | 5698 | 1.35 (1.30-1.41) <0.001 | 1.16 (1.11-1.21) <0.001 | 1.09 (1.05-1.14) <0.001 |
| Q2 | 4628 | 1.08 (1.04-1.13) <0.001 | 1.02 (0.98-1.06) 0.316 | 0.99 (0.95-1.04) 0.754 |
| Q4 | 4764 | 1.11 (1.06-1.15) <0.001 | 1.11 (1.07-1.16) <0.001 | 1.12 (1.07-1.17) <0.001 |

Note: HR: hazard ratio, 95% CI: 95% confidence interval,

Model 1: Crude model. Model 2: Adjusted for sex and age groups. Model 3: Adjusted for sex, age groups, marital status, BMI, education level, smoking status, drinking status, PA, antihypertensive medication, and antidiabetic medication.

BMI: Body mass index, PA: Physical activity.

**Table S14 Sensitivity analysis: Associations of NHHR quartiles with mortality after excluding participants from the first 2 years of follow-up (N = 412259).**

| **Variables** | **Number of deaths** | **Model 1,**  **HR (95% CI) *P* value** | **Model 2,**  **HR (95 % CI) *P* value** | **Model 3,**  **HR (95% CI) *P* value** |
| --- | --- | --- | --- | --- |
| **All-cause mortality** | | | | |
| Q3 | 6893 | Ref | Ref | Ref |
| Q1 | 9205 | 1.36 (1.32-1.40) <0.001 | 1.20 (1.16-1.24) <0.001 | 1.13 (1.09-1.16) <0.001 |
| Q2 | 7556 | 1.10 (1.07-1.14) <0.001 | 1.05 (1.02-1.09) 0.003 | 1.02 (0.99-1.06) 0.166 |
| Q4 | 7414 | 1.08 (1.04-1.11) <0.001 | 1.07 (1.04-1.11) <0.001 | 1.08 (1.04-1.11) <0.001 |
| **Cardio-cerebrovascular disease mortality** | | | | |
| Q3 | 3146 | Ref | Ref | Ref |
| Q1 | 4061 | 1.31 (1.25-1.38) <0.001 | 1.13 (1.08-1.19) <0.001 | 1.08 (1.03-1.14) <0.001 |
| Q2 | 3373 | 1.08 (1.03-1.13) 0.003 | 1.02 (0.97-1.07) 0.458 | 1.00 (0.95-1.05) 0.913 |
| Q4 | 3506 | 1.12 (1.06-1.17) <0.001 | 1.11 (1.06-1.17) <0.001 | 1.12 (1.07-1.17) <0.001 |

Note: HR: hazard ratio, 95%CI: 95% confidence interval,

Model 1: Crude model. Model 2: Adjusted for sex and age groups. Model 3: Adjusted for sex, age groups, marital status, BMI, education level, smoking status, drinking status, PA, antihypertensive medication, and antidiabetic medication.

BMI: Body mass index, PA: Physical activity.

**Table S15 Sensitivity analysis: Associations of NHHR quartiles with mortality after additional adjustment for lipid-lowering medication.**

| **Variables** | **Number of deaths** | **Model 1,**  **HR (95% CI) *P* value** | **Model 2,**  **HR (95 % CI) *P* value** | **Model 3,**  **HR (95% CI) *P* value** |
| --- | --- | --- | --- | --- |
| **All-cause mortality** | | | | |
| Q3 | 9600 | Ref | Ref | Ref |
| Q1 | 13129 | 1.40 (1.36-1.44) <0.001 | 1.22 (1.19-1.26) <0.001 | 1.13 (1.10-1.16) <0.001 |
| Q2 | 10543 | 1.10 (1.07-1.14) <0.001 | 1.05 (1.02-1.08) <0.001 | 1.02 (0.99-1.05) 0.238 |
| Q4 | 10185 | 1.06 (1.03-1.09) <0.001 | 1.06 (1.03-1.09) <0.001 | 1.07 (1.04-1.10) <0.001 |
| **Cardio-cerebrovascular disease mortality** | | | | |
| Q3 | 4363 | Ref | Ref | Ref |
| Q1 | 5762 | 1.35 (1.30-1.41) <0.001 | 1.16 (1.11-1.20) <0.001 | 1.09 (1.04-1.13) <0.001 |
| Q2 | 4669 | 1.08 (1.03-1.12) <0.001 | 1.02 (0.98-1.06) 0.446 | 0.99 (0.95-1.03) 0.552 |
| Q4 | 4796 | 1.10 (1.06-1.15) <0.001 | 1.10 (1.06-1.15) <0.001 | 1.11 (1.07-1.16) <0.001 |

Note: HR: hazard ratio, 95%CI: 95% confidence interval,

Model 1: Crude model. Model 2: Adjusted for sex and age groups. Model 3: Adjusted for sex, age groups, marital status, BMI, education level, smoking status, drinking status, PA, antihypertensive medication, antidiabetic medication and lipid-lowering medication.

BMI: Body mass index, PA: Physical activity

**Table S16 Sensitivity analysis: Associations of NHHR quartiles with mortality after adjustment using WHO BMI classifications.**

| **Variables** | **Number of deaths** | **Model 1,**  **HR (95% CI) *P* value** | **Model 2,**  **HR (95 % CI) *P* value** | **Model 3,**  **HR (95% CI) *P* value** |
| --- | --- | --- | --- | --- |
| **All-cause mortality** | | | | |
| Q3 | 9600 | Ref | Ref | Ref |
| Q1 | 13129 | 1.40 (1.36-1.44) <0.001 | 1.22 (1.19-1.26) <0.001 | 1.14 (1.11-1.18) <0.001 |
| Q2 | 10543 | 1.10 (1.07-1.14) <0.001 | 1.05 (1.02-1.08) <0.001 | 1.02 (0.99-1.05) 0.130 |
| Q4 | 10185 | 1.06 (1.03-1.09) <0.001 | 1.06 (1.03-1.09) <0.001 | 1.07 (1.04-1.10) <0.001 |
| **Cardio-cerebrovascular disease mortality** | | | | |
| Q3 | 4363 | Ref | Ref | Ref |
| Q1 | 5762 | 1.35 (1.30-1.41) <0.001 | 1.16 (1.11-1.20) <0.001 | 1.10 (1.06-1.14) <0.001 |
| Q2 | 4669 | 1.08 (1.03-1.12) <0.001 | 1.02 (0.98-1.06) 0.446 | 0.99 (0.95-1.03) 0.708 |
| Q4 | 4796 | 1.10 (1.06-1.15) <0.001 | 1.10 (1.06-1.15) <0.001 | 1.11 (1.07-1.16) <0.001 |

Note: HR: hazard ratio, 95%CI: 95% confidence interval,

Model 1: Crude model. Model 2: Adjusted for sex and age groups. Model 3: Adjusted for sex, age groups, marital status, BMI (WHO), education level, smoking status, drinking status, PA, antihypertensive medication, and antidiabetic medication.

BMI: Body mass index, PA: Physical activity, WHO: World Health Organization.

**Figure S1 Scaled Schoenfeld residual plot for NHHR quantiles in the mortality model**

**
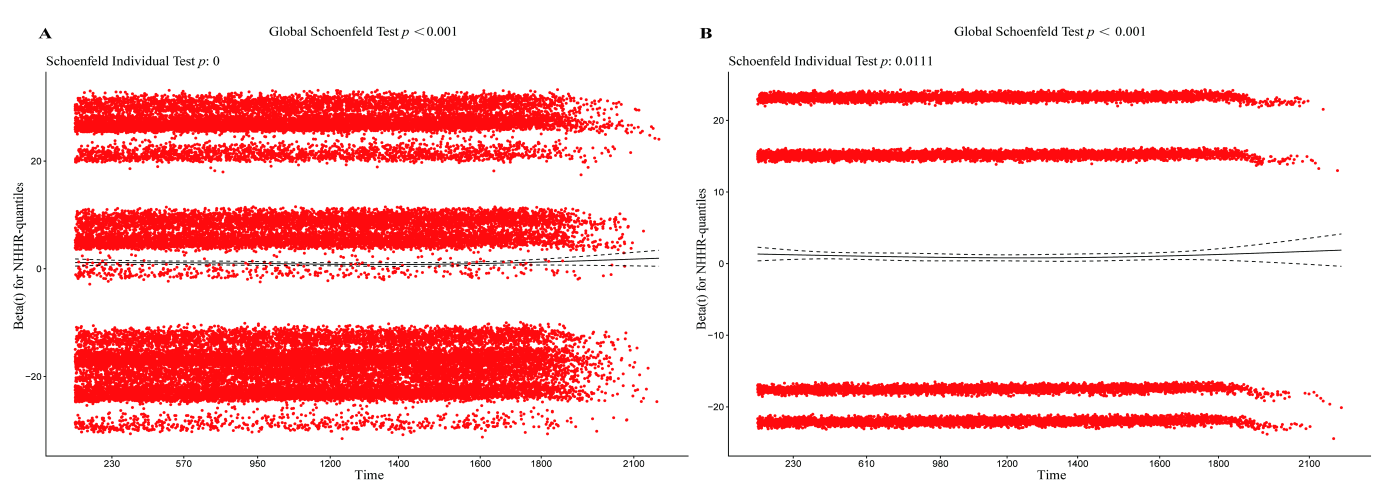
**Note: (A) Scaled Schoenfeld residual plot for NHHR quantiles in the all-cause mortality model; (B) Scaled Schoenfeld residual plot for NHHR quantiles in the cardio-cerebrovascular disease mortality model. The solid line represents the LOESS smoothed fit, with dashed lines indicating the 95% confidence band.

**Figure S2 Restricted cubic spline analyses of the associations between NHHR and mortality across CKM stages 0-3.**


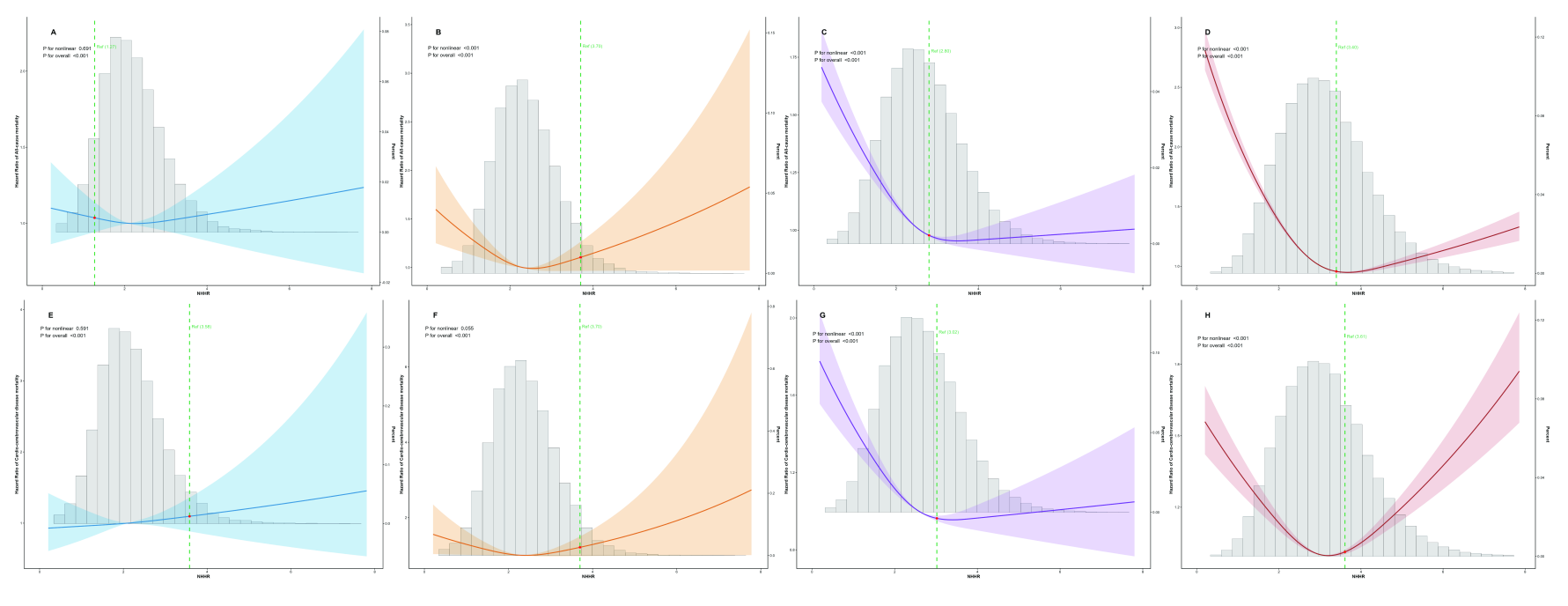


Note: (A) Restricted cubic spline analyses of the associations between NHHR and all-cause mortality across CKM stage 0; (B) Restricted cubic spline analyses of the associations between NHHR and all-cause mortality across CKM stage 1; (C) Restricted cubic spline analyses of the associations between NHHR and all-cause mortality across CKM stage 2; (D) Restricted cubic spline analyses of the associations between NHHR and all-cause mortality across CKM stage 3; (E) Restricted cubic spline analyses of the associations between NHHR and cardio-cerebrovascular disease mortality across CKM stage 0; (F) Restricted cubic spline analyses of the associations between NHHR and cardio-cerebrovascular disease mortality across CKM stage 1; (G) Restricted cubic spline analyses of the associations between NHHR and cardio-cerebrovascular disease mortality across CKM stage 2; (H) Restricted cubic spline analyses of the associations between NHHR and cardio-cerebrovascular disease mortality across CKM stage 3. Adjusted for sex, age groups, marital status, BMI, education level, smoking status, drinking status, PA, antihypertensive medication, and antidiabetic medication. CKM stages: Cardiovascular-kidney-metabolic syndrome stages. BMI: Body mass index, PA: Physical activity.
